# Advancing Sodium-Ion Battery Cathodes: A Low-Cost, Eco-Friendly Mechanofusion Route from TiO_2_ Coating to Ti^4+^ Doping

**DOI:** 10.1021/acs.chemmater.5c01485

**Published:** 2025-08-01

**Authors:** Vadim Shipitsyn, Guanyi Wang, Wenhua Zuo, Ning Zhang, Yongkang Jin, Kangxuan Xia, Cheng Li, Rishivandhiga Jayakumar, Chanmonirath Michael Chak, Yan-Yan Hu, Riqiang Fu, Guiliang Xu, Xianghui Xiao, Jialin Mao, Wenbin Yin, Enyuan Hu, Eric McCalla, Lin Ma

**Affiliations:** † Department of Mechanical Engineering and Engineering Science, The University of North Carolina at Charlotte, Charlotte, North Carolina 28223, United States; ‡ Battery Complexity, Autonomous Vehicle and Electrification (BATT CAVE) Research Center, The University of North Carolina at Charlotte, Charlotte, North Carolina 28223, United States; § Applied Materials Division, 1291 Argonne National Laboratory, 9700 S Cass Avenue, Lemont, Illinois 60439, United States; ∥ Chemical Sciences and Engineering Division, Argonne National Laboratory, 9700 S Cass Avenue, Lemont, Illinois 60439, United States; ⊥ Department of Physics and Atmospheric Science, 3688 Dalhousie University, Halifax B3H 4R2, Canada; # Department of Chemistry and Biochemistry, 7823 Florida State University, Tallahassee, Florida 32306, United States; ¶ Chemistry Division, 8099 Brookhaven National Laboratory, Upton, New York 11973, United States; ∇ Neutron Scattering Division, Oak Ridge National Laboratory (ORNL), Oak Ridge, Tennessee 37831, United States; ○ The National High Magnetic Field Laboratory, Florida State University, Tallahassee, Florida 32310, United States; ⧫ National Synchrotron Light Source II, Brookhaven National Laboratory, Upton, New York 11973, United States; †† 459029 Celgard, LLC, Concord, North Carolina 28027, United States; ‡‡ Department of Chemistry, 5620 McGill University, Montreal, Quebec H3A 0B8, Canada; §§ Department of Applied Physical Sciences, 2331 University of North Carolina, Chapel Hill, North Carolina 27514, United States

## Abstract

Layered oxide battery cathodes often require extra stabilization strategies, such as surface coating or doping, to mitigate side reactions and enhance longevity. Conventional methods such as aqueous deposition and atomic layer deposition are costly and environmentally unfriendly and even damage the original structure, especially for air-sensitive sodium-ion battery (SIB) cathodes. Herein, we introduce an all-dry mechanofusion technique that modifies hydroxide precursors with TiO_2_ coating before sintering with a sodium source. Using advanced characterizations including X-ray diffraction, neutron diffraction, and solid-state nuclear magnetic resonance for structural insights, X-ray absorption spectroscopy to study metal valence states, and transmission X-ray microscopy for nanoscale visualization of nickel oxidation states, we verified that postsintering transforms TiO_2_ surface coating into Ti doping, leading to improved Ni-oxidation homogeneity, modified charge compensation, and enhanced thermal stability. Electrochemical tests reveal superior performance in capacity retention, rate capability, and air stability for these modified cathodes, with pouch cells maintaining over 85% capacity after 650 cycles. This method presents a sustainable, cost-effective route for advanced SIB cathode development.

## Graphical abstract

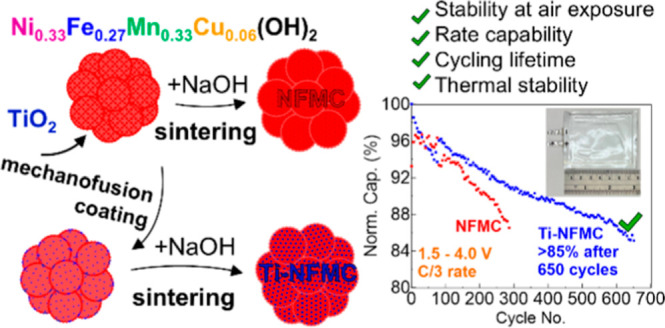

## 1 Introduction

Lithium-ion batteries (LIBs) are pivotal in reducing emissions through the electrification of sectors such as the grid and electric vehicles (EVs).^1,2^ However, the increasing global energy demand highlights the necessity for alternative electrochemical storage solutions to ease the strain on lithium and other scarce metal resources.^3^ Sodium-ion batteries (SIBs) have emerged as viable alternatives, complementing LIBs in the energy storage market due to the plentiful availability of sodium and the development of electrode materials from abundant elements such as carbon, copper, manganese, and iron.^4,5^ Nonetheless, for SIBs to effectively serve in grid energy storage, they must achieve an extremely long lifespan and a relatively high volumetric energy density.

Among the many critical challenges, surface chemistry stands out as pivotal in determining the battery performance of SIBs. Undesired side reactions between electrode materials and electrolytes contribute to capacity fade during extended cycling, particularly when cells are charged to higher voltages for increased energy density.^6^ These reactions can lead to adverse effects, including electrolyte oxidation, gas evolution, reconstruction of the cathode surface, and structural degradation. Surface modification of cathode materials, such as applying an inert protective surface layer (e.g., Al_2_O_3_, TiO_2_),^7,8^ has proven to be an effective strategy to mitigate these side reactions, thereby extending cell lifetime.^6,9^ These coatings not only stabilize the interphase between the cathode and electrolyte but also enhance the cycling stability at high voltages, thereby boosting the cell energy density. Commonly employed methods to constructive these surface layers include atomic layer deposition (ALD)^10,11^ and wet chemistry.^7,12^ ALD ensures a uniform coating but comes with high costs and scaling challenges, while wet chemistry methods generate liquid wastes and require additional processes, such as filtering, drying, and heating.

The mechanofusion method, an innovative, all-dry, and waste-free technique, has recently been recognized for its effectiveness in uniformly coating LIB cathode materials with substances like Al_2_O_3_ ^13^ and for smoothing particle surfaces to decrease surface area.^14^ However, its application to SIB cathodes has been sparingly documented, largely due to the air-sensitive nature of typical SIB layered oxide cathode materials,^15–17^ which can degrade due to sodium loss and structural deformation with prolonged air exposure. This sensitivity poses significant challenges for directly processing these SIB layered oxide cathode materials using the mechanofusion method in an open environment.

In this study, we utilize a commercially viable Ni–Fe–Mn–Cu-based cathode as a model to showcase an effective strategy for modifying SIB layered oxide cathodes through a mechanofusion method. Rather than directly processing air-sensitive SIB cathode materials, we apply the mechanofusion technique to the Ni_0.33_Fe_0.28_Cu_0.06_Mn_0.33_(OH)_2_ precursor, coating it with nano-TiO_2_, followed by solid-state calcination with NaOH as the sodium source. This approach allows us to modify the precursor’s surface before sintering, thereby avoiding prolonged air exposure for the cathodes and preserving the integrity of the cathode material’s performance. To understand the impact of our strategy, we conducted a comprehensive analysis focusing on long-range and local structures, charge compensation mechanisms, and thermal stability. These investigations utilized advanced analytical techniques, including X-ray diffraction (XRD), neutron diffraction (ND), synchrotron-based X-ray absorption spectroscopy (XAS), transmission X-ray microscopy (TXM), scanning transmission electron microscopy (STEM), solid-state nuclear magnetic resonance (ss NMR), and accelerating rate calorimetry (ARC). The results reveal that the TiO_2_ surface coating transforms into Ti^4+^ doping during sintering, forming the O_3_-layered structure NaTi_0.012_Ni_0.326_Fe_0.277_Mn_0.326_Cu_0.059_O_2_ (Ti-NFMC), with no residual TiO_2_ phase. Conversely, sintering the uncoated precursor with NaOH yields O_3_-layered NaNi_0.33_Fe_0.28_Mn_0.33_Cu_0.06_O_2_ (NFMC). In pristine Ti-NFMC, the addition of Ti^4+^ reduces the Ni valence to maintain the charge balance. Nickel serves as the primary redox agent in both Ti-NFMC and NFMC across different states of charge, with Ti-NFMC exhibiting superior chemical homogeneity in Ni-oxidation states and better thermal stability. This study presents a robust and cost-efficient method for surface coating that transitions into structural doping on SIB cathode materials, mitigating the negative impact of moisture during the process, thereby paving a way toward the design of high-performance SIB cathode materials.

## 2 Experimental Section

### 2.1 Material Preparation

Mechanofusion was performed at a spinning speed of 2400 rpm for 60 min following a previous report.^18^ During this process, the Ni_0.33_Fe_0.28_Cu_0.06_Mn_0.33_(OH)_2_ precursor (Hunan Zoomwe Zheng Yuan Advanced Material Trade Co., Ltd.) was coated with a nanosized TiO_2_ powder (Sigma-Aldrich). 50 g of the Ni_0.33_Fe_0.28_Cu_0.06_Mn_0.33_(OH)_2_ precursor was loaded inside the mechanofusion bowl with 0.45 g of TiO_2_ (∼1 wt %). Both uncoated and coated precursors were ground with NaOH (Sigma-Aldrich) according to a 1:1 molar ratio and annealed at 800 °C for 15 h in air with a heating and cooling rate of 5 °C/min. The sintered cathode material was then transferred directly to an Ar-filled glovebox at 300 °C.

### 2.2 Material Characterization

Powder XRD analysis was conducted in-house using a Rigaku Miniflex 600 diffractometer equipped with Cu Kα radiation (wavelength of 1.5406 Å). Cross-sectional specimens were prepared by using a dual-beam focused ion beam–scanning electron microscopy system (Zeiss Nvision40) and a JEOL ion beam cross-section polisher (model IB-0900CP).

Full field three-dimensional (3D) TXM experiments were carried out at the Full Field X-ray Imaging (FXI) beamline 18-ID of the National Synchrotron Light Source II (NSLS-II) at Brookhaven National Laboratory. The reconstruction of TXM data sets was accomplished using a well-built scientific software package, TXM_Sandbox,^19^ available at https://github.com/xianghuix/TXM_Sandbox. Segmentation, quantification, and visualization of the TXM data were conducted using Dragonfly software, version 2022.2, provided by Comet Technologies (available at https://www.theobjects.com/dragonfly).

ND experiments were conducted using the POWGEN instrument at the Spallation Neutron Source (SNS) located at the Oak Ridge National Laboratory. About 1 g of powder was encapsulated in a 6 mm diameter vanadium can for the POWGEN Automated Changer (PAC). Diffraction data was gathered at 293 K over approximately 2 h, followed by routine data reduction procedures. The center wavelength was 0.8 Å, which enabled coverage of a *d*-spacing range from 6.2 to 0.1 Å. Solid-state NMR ^23^Na Magic-Angle-Spinning (MAS) solid-state NMR experiments were performed using a Bruker Avance-II 300 MHz spectrometer at a ^23^Na Larmor frequency of 158 MHz. The sample was packed into 4 mm rotors in an argon-filled glovebox and spun at 10 kHz for NMR measurements. The pjMATPASS pulse sequence was employed to achieve high-resolution NMR spectra.^20^ The recycle delay was 0.1 s, and the 90° pulse length was 4.43 μs.

The ARC analysis was performed with an MMC 274 Nexus calorimeter (NETZSCH). Two pieces of (4.0 V vs Na^+^/Na at C/20-rate) electrodes (ϕ 11 mm) charged in two-electrode cell configuration (with Na metal) were extracted and sealed with 25 mg of 1 m NaPF_6_ (>99%, CapChem Technology) in propylene carbonate (PC, H_2_O <20 ppm, CapChem Technology) + 5 wt % fluoroethylene carbonate (FEC, H_2_O <20 ppm, CapChem Technology) electrolyte into a stainless steel tube using an inert gas welding machine inside an Ar-filled glovebox. The ARC response was tracked from 50 to 300 °C temperature range under adiabatic conditions, when the self-heating rate (SHR) exceeded 0.03 °C/min.

STEM characterization was carried out by an FEI Talos F200X. In addition to imaging, energy-dispersive X-ray spectroscopy (EDS) was employed to analyze the elemental composition and distribution. The transmission electron microscopy (TEM) samples were prepared by using focused ion beam scanning electron microscopy (FIB-SEM, Zeiss NVision 40) through a standard lift-out and milling procedure. First, a small region (2 × 20 μm) of interest was identified under the SEM, and a protective carbon layer was deposited on the sample to prevent damage from the ion beam. FIB was then used to mill away the surrounding material, creating a lamella (a thin slice of the sample). This lamella was lifted with a tungsten probe and transferred onto a TEM grid. The final thickness of the TEM lamella was approximately 100 nm.

The X-ray absorption spectroscopy (XAS) spectra for the Mn (6539 eV), Fe (7112 eV), Ni (8333 eV), and Cu (8979 eV) K-edges were collected at the 7-BM (QAS) beamline of the National Synchrotron Light Source-II (Brookhaven National Lab (BNL), USA) in transmission mode. Mn, Fe, Ni, and Cu metal foils were used as the standard references to calibrate energy shifts. MnSO_4_·H_2_O (99% Thermo Fischer Scientific), MnO_2_ (99.9% Thermo Fischer Scientific), FeSO_4_·7H_2_O (98% Thermo Fischer Scientific), Fe_2_O_3_ (99.9% Thermo Fischer Scientific), NiSO_4_·6H_2_O (99% Thermo Fischer Scientific), NiCO_3_ (anhydrous, 98% Thermo Fischer Scientific), CuSO_4_·5H_2_O (98% LabChem), and CuO (99% Thermo Fischer Scientific) were used as references of the transition metal oxidation states. These references were prepared by mixing sucrose (C_12_H_22_O_11_) with the chemicals containing transition metal in the needed stable oxidation state to achieve the mixture with 5 wt % of the studied metal. Mixed chemicals were pelletized (0.1 g) and sealed in Kapton film to prevent influence of air environment. Electrodes (active material, binder, carbon black in the 92:4:4 wt % ratio) on the Al foil (ϕ 11 mm with ∼8.0 mg cm^–2^ average active material loading) of NFMC and Ti-NFMC materials were prepared in different states of charge. Three cells for each electrode material charged to 3.2 and 4.0 V and discharged to 2.0 V (after charging to 4.0 V) vs Na^+^/Na in CC­(C/10)–CV­(C/100) mode were recovered from the two-electrode cells (with Na metal) in an Ar-filled glovebox and sealed in Kapton film. Four spectra were collected for each K-edge and merged to improve the signal-to-noise ratio. Athena software program package was used to analyze X-ray absorption near edge structure (XANES) spectra. Background, pre-edge and postedge lines were defined to normalize XANES spectra. The extended X-ray absorption fine structure (EXAFS) spectra were Fourier transformed in the 3.0–13.7 Å^–1^ *k*-range. Fitting was done with the 1.0–4.0 Å *R*-range using the Artemis software program package. Passive electron reduction factor S_o_^2^, Debye–Waller factor σ^2^, scattered distances *R*, and the Δ*E*_o_ parameter were included in the math expression for refinement. Refined structure of the electrode material from the XRD pattern was used for the EXAFS fitting. To simplify the model for the fitting, only the core element was used to calculate TM–O, TM–TM, and Na–TM single scattering paths length and other unique double scattering paths.

### 2.3 Electrochemical Measurements

NFMC and Ti-NFMC electrodes were prepared for electrochemical testing by mixing the active material with an acetylene black conductive agent (Timcal C45) and poly­(vinylidene) (PVDF, Solef 5130) in a weight ratio of 92:4:4. The mixture was dissolved in *N*-methyl pyrrolidone (NMP) to form a viscous slurry. The obtained slurry was spread onto Al foil using a Dr. Blade and then dried in a glovebox antechamber under vacuum overnight. The punched electrodes (ϕ 11 mm with ∼8.0 mg cm^–2^ average active material loading for half-cells and 26 × 42 mm with ∼5.4 mg cm^–2^ average active material loading for single-layer pouch (SLP) cells) were dried at 110 °C for 12 h under vacuum overnight. CR-2032 coin cells coupled with a Na metal anode were tested using 1 m NaPF_6_ in PC + 5 wt % FEC electrolyte with glass fiber separators (Whatman GF/A) in the voltage range 2.0–4.0 V vs Na^+^/Na. The cycling performance in SLP was performed versus a hard carbon anode (27 × 43 mm) with a N/P ratio of ∼1.05 using 1 m NaPF_6_ in PC + 2 wt % 1,3,2-dioxathiolane 2,2-dioxide (DTD, H_2_O <20 ppm, CapChem Technologies) electrolyte with a PC wettable separator (Celgard) in the voltage range 1.5–4.0 vs HC. This separator, which is compatible with PC, is a 12 μm thick PP coated with 0.5 μm PVDF on both sides using a special treatment for full wettability with PC.

## 3 Results and Discussion

Figure 1a shows a schematic representation of the dry-particle fusion process used for the surface coating of TiO_2_ (Figure S1) onto the Ni_0.33_Fe_0.28_Cu_0.06_Mn_0.33_(OH)_2_ precursor (Figures 1b, S2, and S3). Central to the apparatus is a rotating bowl, approximately 10 cm in diameter, equipped with a stationary hammer and scraper. The coating material and target powder are loaded into the bowl. As the bowl rotates, the hammer compacts the powders against the bowl wall, while the scraper detaches the material from the wall, preparing it for subsequent cycles under the hammer. Prior to the dry-particle fusion process, the precursor exhibits a spherical and dense morphology with particle sizes of approximately 5 μm (Figure 1b). Remarkably, after the TiO_2_ surface coating through dry-particle fusion, the particle morphology remains largely unchanged (Figures 1c, S4, and S5), now featuring a uniform TiO_2_ layer on the surface (Figures S4 and S5).

**Figure 1:**
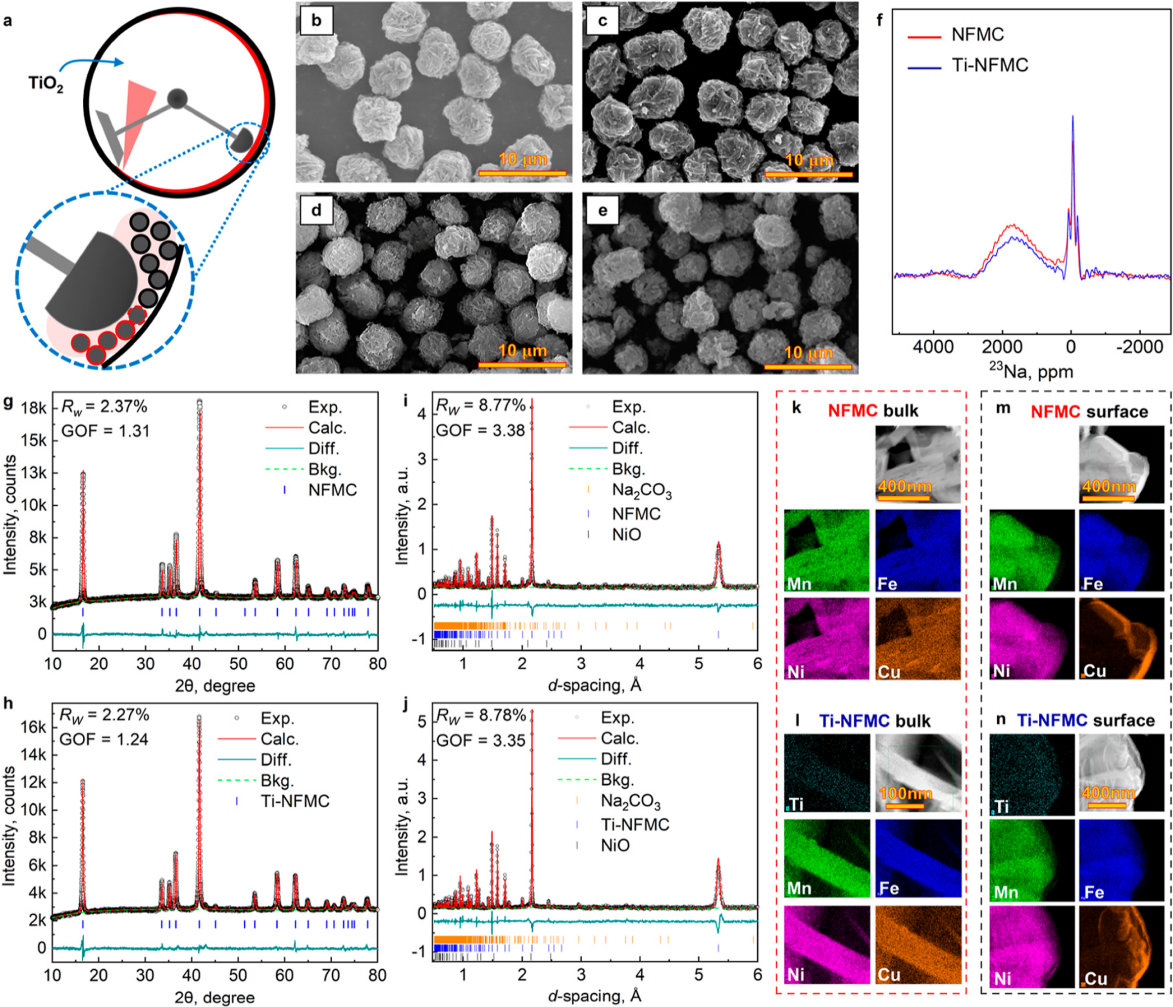
Synthesis of NFMC and Ti-NFMC materials. (a) A schematic of the mechanofusion method for processing precursors. SEM images of the (b) NFMC precursor (i.e., Ni_0.33_Fe_0.28_Cu_0.06_Mn_0.33_(OH)_2_); (c) Ti-NFMC precursor (i.e., Ni_0.33_Fe_0.28_Cu_0.06_Mn_0.33_(OH)_2_ coated with 1 wt % TiO_2_); (d) NFMC sintered from (b) with NaOH at 800 °C; and (e) Ti-NFMC sintered from (c) with NaOH at 800 °C. (f) ^23^Na-projected pjMATPASS spectra of NFMC and Ti-NFMC. XRD of (g) NFMC and (h) Ti-NFMC. ND of (i) NFMC and (j) Ti-NFMC. TEM–EDX of (k,m) NFMC and (l,n) Ti-NFMC.

After sintering at the optimized conditions (800 °C in air), both sodiated compounds (Table S1), NFMC (Figure 1d) and Ti-NFMC (Figure 1e), exhibit similar spherical morphologies. The optimization of sintering conditions was based on the interplay between the sintering temperature and the molar ratio of NaOH to precursor (Figures S6 and S7). Insufficient sintering temperature results in inadequate solid-state reactions and poor crystallinity (Figure S6a,b), whereas excessively high temperatures promote the formation of NiO impurities (Figure S6c), which is consistent with previous reports.^21–23^ A higher NaOH-to-precursor molar ratio leads to residual Na_2_CO_3_ after sintering (Figure S7). Therefore, a sintering temperature of 800 °C and a NaOH-to-precursor molar ratio of 1:1 were chosen as the optimal conditions for this study. Detailed characterizations were performed on the pristine materials sintered from both coated and uncoated precursors to assess the differences under the optimized sintering conditions. ssNMR, XRD, and ND were first employed to investigate both the local and long-range structures of NFMC and Ti-NFMC, respectively (Figure 1f–j). The powder XRD patterns (Figure 1g–h) confirm that both samples exhibit a single-phase structure, which can be indexed to the *R*3̅*m* space group, characteristic of the α-NaFeO_2_ structure. Notably, no TiO_2_ peaks are observed in the pattern (Figure 1h), indicating that Ti has been fully incorporated into the cathode material through the solid-state reaction. This observation is consistent with studies reporting the thermally activated migration of surface-deposited Ti into the bulk during high-temperature sintering. Mechanisms such as solid-state diffusion, bulk (lattice) diffusion, and recrystallization are known to promote incorporation of Ti from the particle surface into the bulk structure at elevated temperatures. While the detailed kinetics and thermodynamics of these processes merit further investigation, they are beyond the scope of this study. The crystal structures of the samples were further refined using the General Structure Analysis System (GSAS II) software,^24^ based on the initial structural model of α-NaFeO_2_. The refined crystallographic data summarized in Table S2 indicate that Ni, Mn, Fe, Cu, and Ti ions occupy the same Wyckoff sites, confirming the uniform incorporation of Ti^4+^ into the transition-metal oxide layer. Ti^4+^ doping results in a slight increase in the lattice parameters (a and c, Table S2), which can be attributed to the varying ionic radii of Ni^2+^ (0.69 Å), Ni^3+^ (0.56 Å), Fe^3+^ (0.645 Å), Mn^4+^ (0.54 Å), and Ti^4+^ (0.604 Å).^25–27^

The O_3_-type structure of NFMC and Ti-NFMC, belonging to the *R*3̅*m* space group, was confirmed through the ND pattern and subsequent Rietveld refinement (Figure 1i–j and Table S3). The refined lattice parameters are *a* = 2.9744(1) Å and *c* = 15.9998(6) Å for NFMC, and *a* = 2.9751(5) Å and *c* = 16.0079(4) Å for Ti-NFMC. A slight increase in lattice parameters was observed, consistent with results from XRD refinements. ND, which relies on the coherent scattering length of atoms rather than their atomic number, enables the identification of ordering between transition metals. Rietveld refinement of the ND data suggests that transition metals (Ti, Fe, Ni, Cu, and Mn) are distributed randomly, as the structural model with random transition metal distribution closely matches the observed pattern (Figure 1i–j), and no additional superlattice reflections were observed. Therefore, long-range in-plane transition metal ordering appears unlikely. Refinement is also utilized to identify potential impurities, including NiO (formed during sintering) and Na_2_CO_3_ (generated during sample transportation due to air sensitivity). For NFMC, the refined composition indicates 97 wt % NFMC, 0.8 wt % NiO, and 2.2 wt % Na_2_CO_3_, respectively. In contrast, for Ti-NFMC, the composition is refined to 98.1 wt % Ti-NFMC and 1.9 wt % Na_2_CO_3_ without the NiO impurity. The formation of an ionically and electronically resistive Na_2_CO_3_ layer on the cathode surface typically increases interfacial resistance and degrades the kinetic performance, while the formation of electrochemically inactive NiO reduces the specific capacity of cathode active materials. Ideally, these should be minimized on the cathode surface for practical applications.

Ex situ ^23^Na MAS NMR spectroscopy offers a detailed examination of the local environment surrounding sodium ions in the layered structures of both NFMC and Ti-NFMC (Figure 1f). The large ^23^Na NMR shifts, observed at approximately 2100 ppm for both samples (Figures 1f and S8), are attributed to the hyperfine interactions between Na and the paramagnetic transition metal centers (Fe, Mn, Ni, Cu) within the materials,^28^ as illustrated by the deconvolution analysis (Figure S9). The introduction of Ti^4+^ doping does not appear to significantly alter the main peak position of sodium (Figures 1f and S8) at around 2100 ppm. Nevertheless, our analysis indicates that Ti^4+^ doping increases the disorder of transition metal arrangement around Na^+^ (Figure S9) indicated by the increased intensity in the shift range between 500 and 1500 ppm, suggesting successful Ti^4+^ doping. Additionally, a small and narrow signal near 0 ppm (Figures 1f and S8) can likely be attributed to the presence of diamagnetic species, such as Na_2_CO_3_,^29^ which aligns with observations from ND data (Figure 1i–j). The incorporation of Ti^4+^ doping also enhances the proportion of these diamagnetic species (Figure S9). STEM combined with EDX spectroscopy was employed to analyze NFMC and Ti-NFMC samples prepared via FIB techniques. Both the bulk (Figure 1k–l) and surface (Figure 1m–n) regions were examined. The analysis suggests that all elements, including Ni, Mn, Cu, and Fe, are uniformly distributed throughout the entire particle of NFMC. Similarly, for Ti-NFMC, all elements—Ni, Mn, Cu, Fe, and Ti—are uniformly distributed across the entire particle.

To investigate the impact of Ti^4+^ doping on the transition metal valence states in pristine NFMC and Ti-NFMC, the normalized XANES spectra at the Mn K-edge, Fe K-edge, Ni K-edge, and Cu K-edge, along with reference compounds, were then utilized (Figure 2a). Two key features are observed in the normalized XANES spectra for 3d transition metal elements in layered structures: the pre-edge peak at lower energy, attributed to the dipole-forbidden 1s → 3d transition, and the main edge peak at higher energy, corresponding to the 1s → 4p transition.^30,31^ The Mn K-edge spectra (Figure 2a) exhibit a main edge position identical to that of MnO_2_ for both NFMC and Ti-NFMC, indicating that Mn ions are predominantly present as Mn^4+^. Similarly, the Fe K-edge spectra (Figure 2a) show a main edge position matching that of Fe_2_O_3_, suggesting that Fe ions are mainly Fe^3+^. For Ni ions (Figure 2a), the main edge for NFMC is located at ∼8351.5 eV, indicating a mix of Ni^2+^ and Ni^3+^ ions within the structure.^30^ In contrast, the main edge for Ti-NFMC shifts toward lower photon energy, suggesting the average Ni valence approaches ∼+2, likely due to charge neutralization induced by Ti^4+^ doping. Lastly, the Cu K-edge spectra (Figure 2a) align with those of CuSO_4_, showing that Cu ions in both NFMC and Ti-NFMC are predominantly Cu^2+^.

**Figure 2:**
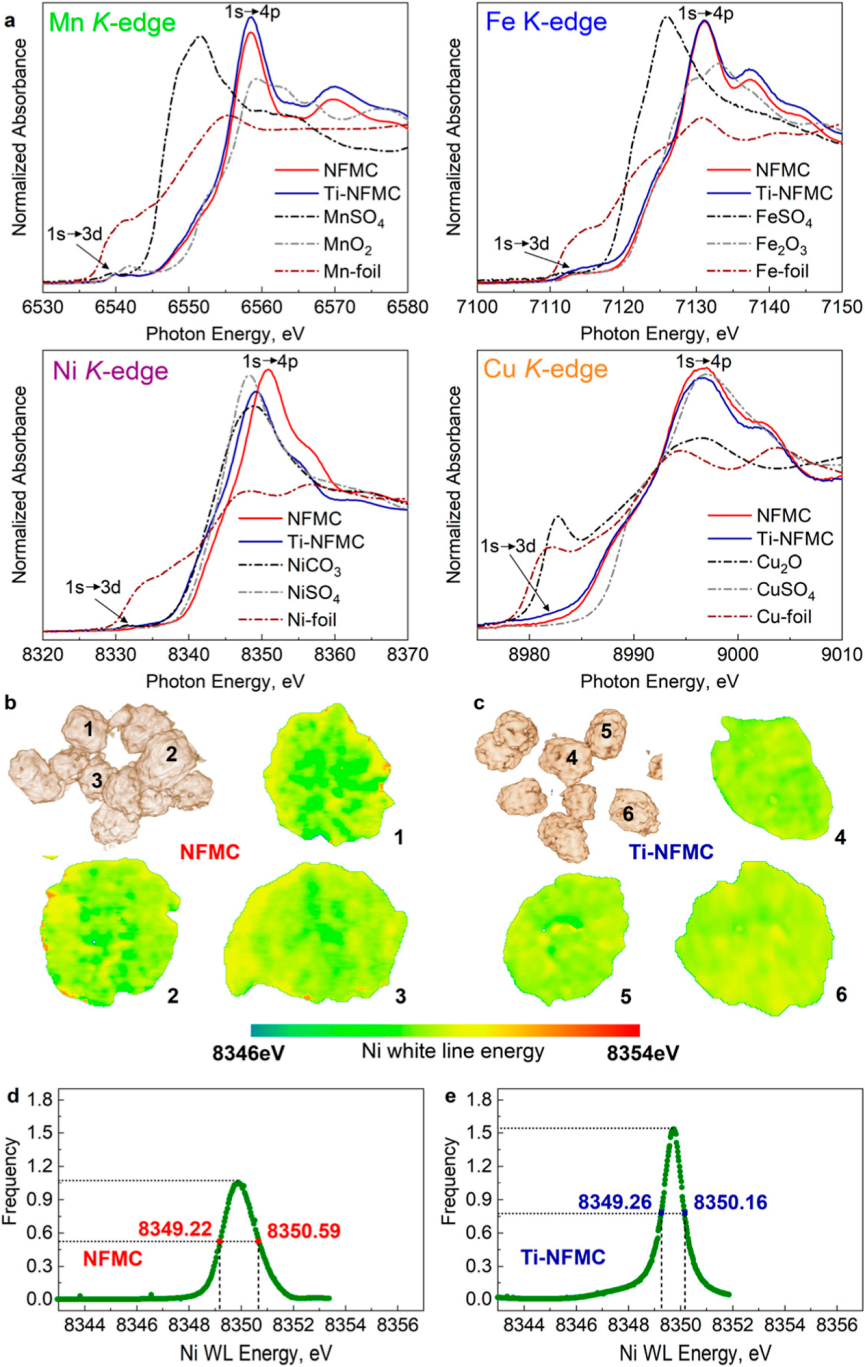
Characterizations of the valence of pristine NFMC and Ti-NFMC materials. (a) XANES of Mn, Fe, Ni, and Cu in NFMC and Ti-NFMC compared with reference samples. (b) 3D TXM-XANES results and corresponding 2D rendering of chemical mapping for Ni of (b) NFMC and (c) Ti-NFMC. (d) Ni K-edge white-line energy histograms of NFMC (average results of particles 1–3) and Ti-NFMC (average results of particles 4–6).

To complement the XANES results across different length scales, we conducted 3D Ni K-edge full-field TXM on both pristine NFMC (Figure 2b) and Ti-NFMC (Figure 2c). This approach allowed us to visualize the evolution and distribution of Ni-oxidation states on the secondary particle scale. The choice of TXM was motivated by the clear valence differences between NFMC and Ti-NFMC observed in XANES (Figure 2a) and its capability to detect local chemical homogeneity at the particle level by analyzing the absorption spectrum of specific elements with a nominal spatial resolution of ∼30 nm.^32,33^ The NFMC particles exhibited a higher valence state of Ni (higher white-line energy) and more heterogeneity in Ni-oxidation states, indicating a higher level of oxidation. Conversely, Ti-NFMC particles displayed a uniform distribution of nickel valence states, suggesting more consistent Ni-oxidation throughout the sample. To quantify this chemical heterogeneity, we collected white-line energy histograms for both materials.^34^ The full width at half-maximum (fwhm) of the histogram for NFMC particles (1.37 eV, Figure 2d) was obviously broader than for Ti-NFMC (0.9 eV, Figure 2e), indicating greater variability in Ni-oxidation states. The narrower spectral distribution and higher concentration of spectral intensity in the central energy range for Ti-NFMC suggest that this material achieves a higher degree of chemical homogeneity regarding the Ni-oxidation state.

Ti^4+^ doping has been widely recognized and documented in many studies for its effectiveness in enhancing the electrochemical performance of cathodes by mitigating phase transitions induced by the Jahn–Teller effect and facilitating sodium-ion transport kinetics.^35–38^ In this study, electrochemical performance tests were conducted to demonstrate the benefits of our Ti^4+^ doping strategy in sodium-ion cathode materials (Figure 3). The electrochemical performance of the NFMC and Ti-NFMC cathodes was initially assessed in coin cells paired with a Na metal anode at room temperature (25 °C) over a voltage range of 2.0–4.0 V (Figures 3a–d, S10). An electrolyte solution of 1 m NaPF_6_ in PC with 5 wt % FEC was chosen due to FEC’s proven effectiveness in enhancing the reversibility of the Na metal anode,^39,40^ which supports this demonstration. At a cycling rate of C/2, the Ti-NFMC cathode exhibited superior capacity retention, maintaining ∼85% of its capacity after ∼80 cycles (Figures 3a–b, S10). In contrast, the specific capacity of the NFMC sample dropped to around 100 mAh g^–1^ after 40 cycles (Figure 3a), while the Ti-NFMC sample retained a specific capacity of ∼120 mAh g^–1^ (Figure 3b). To further evaluate the electrochemical performance of the prepared materials, their rate capabilities were assessed, as shown in Figure 3c. The cells underwent charge and discharge cycles at various C rates, as indicated. The Ti-NFMC exhibited superior rate capability and stable capacities across all tested C rates (Figure 3c). At 0.1C, the initial discharge capacity of Ti-NFMC was approximately 140 mAh g^–1^. Remarkably, the electrode retained ∼96%, ∼94%, ∼91%, ∼89%, ∼87%, ∼83%, ∼77%, ∼64%, and ∼55% of its initial capacity when the C rate increased to 0.4C, 0.6C, 1.3C, 2C, 2.5C, 3.8C, 6.4C, and 12.5C, respectively. Upon returning to 0.1C, the capacity was fully recovered, highlighting the structural stability of the electrode under high current density conditions. Relationship between the cycling performance and the material structure could be the main reason for the superior performance of Ti-NFMC. Rietveld refinement confirmed that Ti-doping increased the lattice parameters causing enlarged *d* spacing and a wider Na-ion diffusion layer, thereby facilitating Na-ion diffusion during cycling, resulting in easier Na diffusion within cycling. Moreover, according to ND results, higher amount of Na_2_CO_3_ and NiO impurities in the NFMC cathode could affect the de/intercalation of Na within cycling due to possibly higher impedance. Furthermore, TXM-XANES analysis demonstrated a higher degree of chemical homogeneity in Ti-NFMC, which could be critical for enhancing the long-term battery cycling performance.

**Figure 3:**
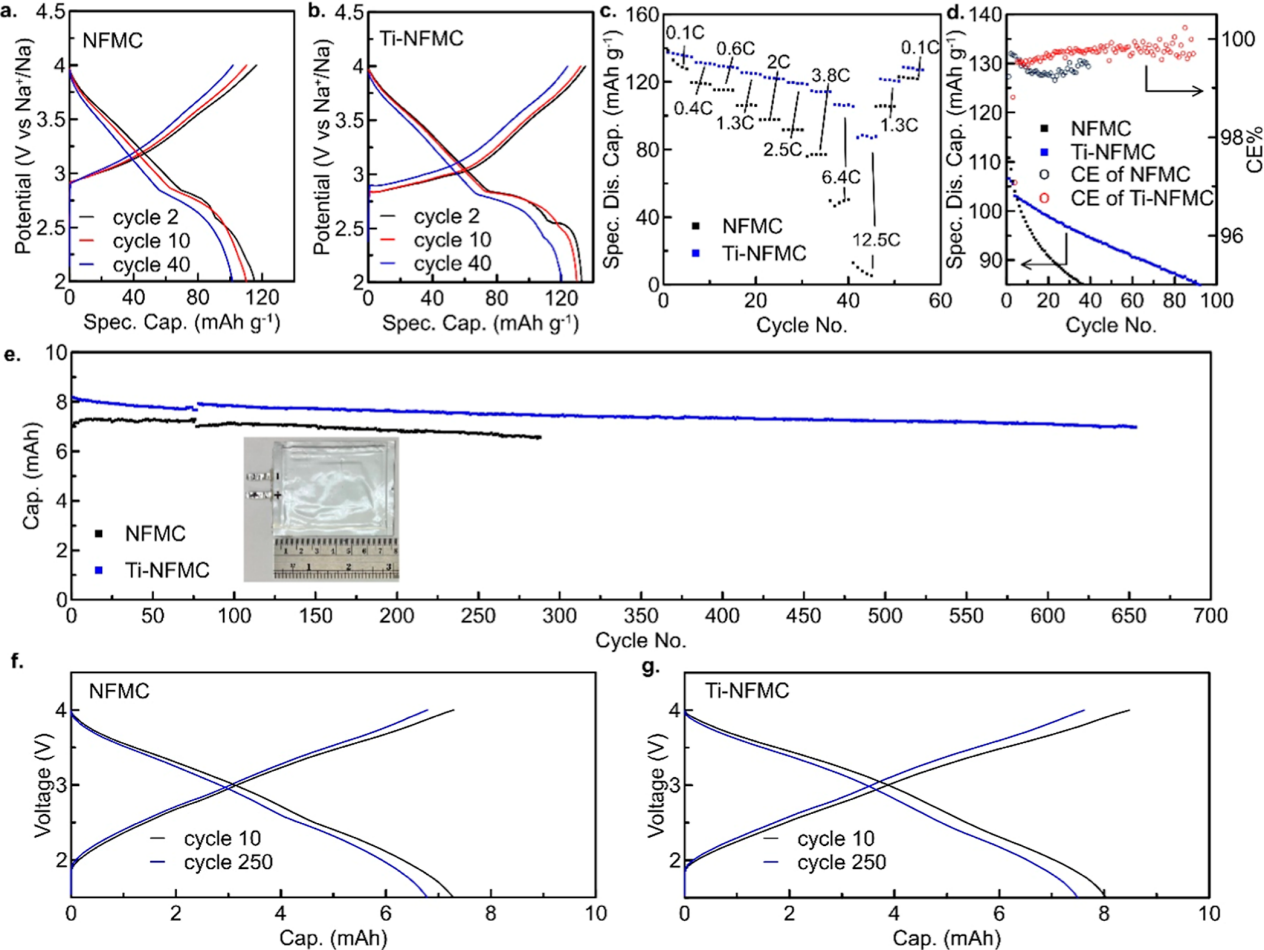
Electrochemical evaluation of NFMC and Ti-NFMC materials using both coin cells and single-layer pouch cells. Charge–discharge curves of (a) NFMC and (b) Ti-NFMC cathodes coupled with a Na metal anode in coin cells in the voltage range 2.0–4.0 V at 0.5C and 25 °C with 1 m NaPF_6_ in PC + 5 wt % FEC. (c) Specific discharge capacity vs cycle number for NFMC and Ti-NFMC using different rates as labeled in the voltage range 2.0–4.0 V at 25 °C with 1 m NaPF_6_ in PC + 5 wt % FEC. (d) Cycling stability of NFMC and Ti-NFMC cathodes after 48 h air exposure, coupled with a Na metal anode in coin cells, in the voltage range 2.0–4.0 V at 25 °C with 1 m NaPF_6_ in PC + 5 wt % FEC. (e) Cycling stability of NFMC and Ti-NFMC cathodes, coupled with a hard carbon anode in single-layer pouch cells, in the voltage range 1.5–4.0 V at C/3 and 25 °C with 1 m NaPF_6_ in PC + 2 wt % DTD. Charge–discharge curves of (f) NFMC and (g) Ti-NFMC at different cycles corresponding to the pouch cells in (e).

Air stability is another crucial aspect of sodium-ion-layered oxides, as air-sensitive layered oxides require protection from moist environments to preserve their structure and stoichiometry, leading to increased manufacturing and storage costs. In this study, the Ti-NFMC cathode demonstrated better capacity retention after 48 h of air exposure (50% relative humidity), maintaining ∼85% of its capacity after ∼60 cycles, in comparison to the NFMC sample (Figure 3d). This aligns with previous findings that highlight the benefits of Ti^4+^ doping in enhancing air stability.^41^ The detailed mechanisms underlying improved air stability may involve reduced reactions with adsorbed water, preserved structural stability, and minimal changes in chemical composition during exposure.^16^ However, the detailed analysis of these mechanisms is complex and beyond the scope of this work and thus will not be discussed further. Additionally, air exposure led to specific capacity loss in both NFMC and Ti-NFMC (Figure 3a,b,d), consistent with previous reports,^42^ highlighting the need for further advancements to address air stability challenges in SIB layered oxide cathodes.

To further demonstrate the practical applicability of the Ti-NFMC cathode, lab-scale pouch cells with a single-side coating (∼10 mAh) were fabricated and tested at C/3 and room temperature (25 °C) as a proof of concept (Figures 3e–g and S11). The cells incorporated a hard carbon anode with an N/P ratio of ∼1.05 and utilized 1 m NaPF_6_ in PC with 2 wt % DTD as the electrolyte. The addition of DTD was selected based on its proven effectiveness as a state-of-the-art additive for sodium-ion batteries with layered oxide cathodes and hard carbon anodes.^43^ Pouch cells utilizing the Ti-NFMC cathode material demonstrate prolonged cycling stability, retaining >85% of their capacity after 650 cycles (Figures 3e, S11).

To further understand the effect of Ti^4+^ doping on the properties of electrochemically charged particles, we use XANES, EXAFS, TXM, and ARC to investigate NFMC and Ti-NFMC from the perspective of charge compensation mechanisms, local structural changes, nickel oxidation state distribution, and thermal stability at elevated temperatures. To understand the charge compensation mechanisms at different states of charge, ex situ XANES was performed for samples charged to 3.2 V, 4.0 V, and discharge back to 2.0 V using C/10 at 40 °C during the first cycle (Figure S12). The Mn K-edge spectra (Figure 4a–b) exhibit a main edge position identical with that of MnO_2_ for both NFMC and Ti-NFMC at different voltages, indicating that Mn ions are predominantly present as Mn^4+^ and do not take part in the electrochemical redox reaction. The Fe K-edge spectra (Figure 4a–b) show a main edge position matching Fe_2_O_3_ at 3.2 V and discharge back to 2.0 V, suggesting that Fe ions are mainly Fe^3+^ at 3.2 V and discharge back to 2.0 V. But it slightly shifts higher when charging to 4.0 V, suggesting its valence increase at 4.0 V. For Ni ions (Figure 4a–b), the higher the voltage, the further the main edge shift for both NFMC and Ti-NFMC, suggesting Ni is the major redox element for both samples. Lastly, the Cu K-edge spectra (Figure 4a–b) align with CuSO_4_ at different voltages, showing that Cu ions in both NFMC and Ti-NFMC are predominantly Cu^2+^ at different voltages here and do not take part in the electrochemical redox reaction. The detailed local structural change was further analyzed via corresponding EXAFS spectra at Mn, Fe Ni, and Cu K-edges, as shown in Figure S13 and Table 4. The spectrum of both NFMC and Ti-NFMC materials is in good agreement with that of the layered structure, where the first peak arises from the backscattering of oxygen anions around 3d transition metal ions and the second-shell peak is mainly due to the scattering from the metal ions.^30^ It can be observed that the Mn–O and Cu–O bond lengths are not affected during the charge and discharge processes for both NFMC and Ti-NFMC samples (Table S4), which is in good agreement with the XANES results (Figure 4a–b) and again proves the presence of inactive Mn^4+^ and Cu^2+^ in this structure. Conversely, the Fe–O and Ni–O bond lengths in these materials decrease as charging of the pristine material to higher voltages due to the oxidation of Fe and Ni ions; however, the Fe–O bond length in Ti-NFMC does not follow this decreasing trend. After discharging, these bond lengths largely return to their initial values (Table S4).

**Figure 4:**
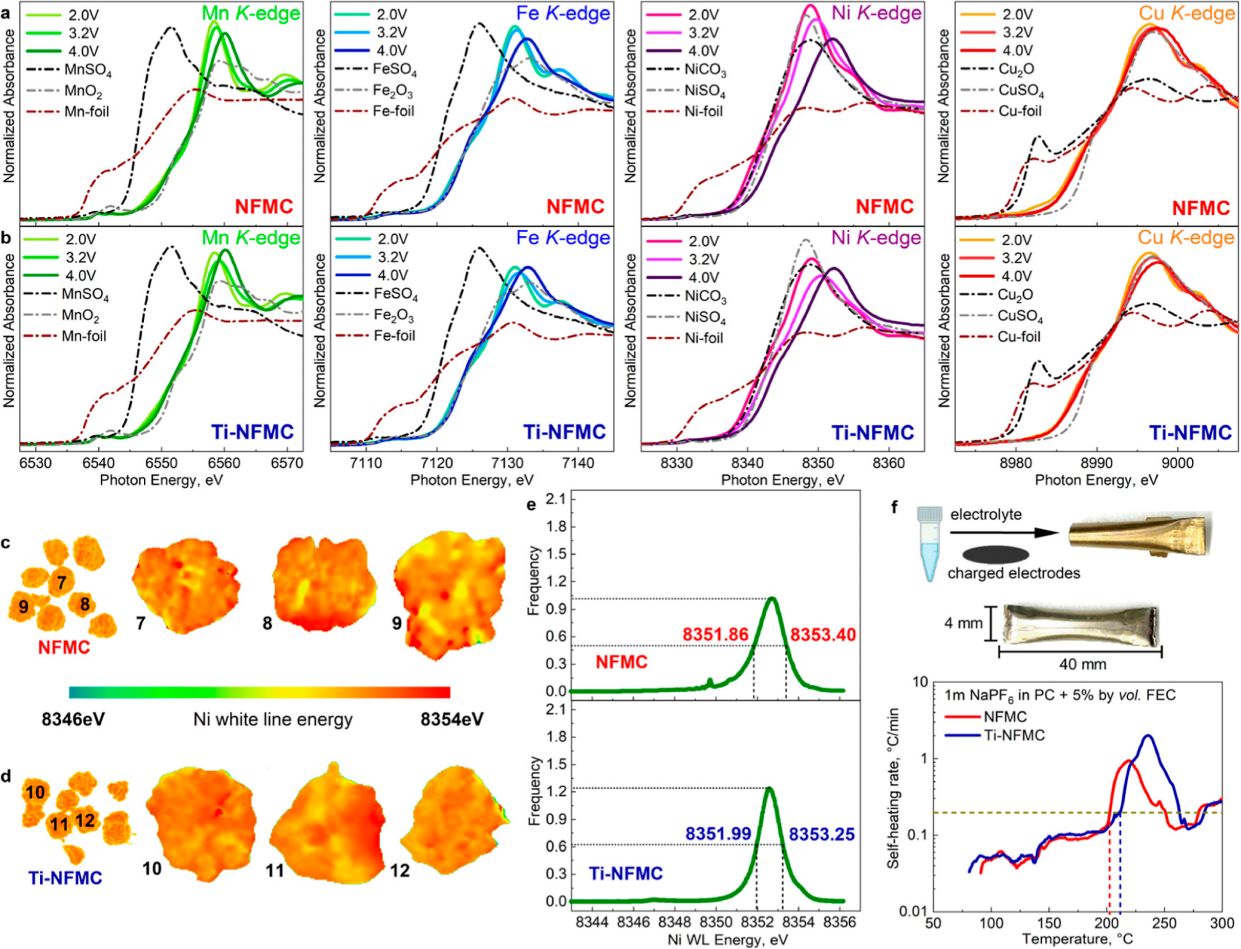
Characterizations of electrochemically charged NFMC and Ti-NFMC materials. XANES of Mn, Fe, Ni, and Cu in (a) NFMC and (b) Ti-NFMC compared with reference samples; 3D TXM-XANES results and corresponding 2D rendering chemical mapping for Ni of (c) charged NFMC (4.0 V vs Na^+^/Na) and (d) charged Ti-NFMC (4.0 V vs Na^+^/Na); (e) Ni K-edge white-line energy histograms of charged NFMC (average results of particles 7–9) and charged Ti-NFMC (average results of particles 10–12); (f) self-heating rate vs temperature for reactions between charged NFMC and Ti-NFMC (4.0 V vs Na^+^/Na) with electrolytes at elevated temperatures.

To complement the XANES results at various scales, we utilized 3D Ni K-edge full-field TXM to analyze the charged states of NFMC (Figure 4c) and Ti-NFMC (Figure 4d) at 4.0 V. This approach allowed us to visualize the distribution of Ni-oxidation states at a high state of charge at the secondary particle scale. We noted a shift in the white-line energy to approximately 8352.5 eV. Similar to pristine samples, the charged NFMC particles exhibited a mosaic-like heterogeneity in Ni-oxidation states, particularly noticeable as yellow regions on both the particle surfaces and bulk, indicating areas lacking full oxidation. In contrast, Ti-NFMC particles presented a more uniform distribution of nickel valence states and a higher degree of chemical homogeneity (Figure 4e). The reactivity of charged NFMC and Ti-NFMC with electrolyte at elevated temperatures was characterized by ARC. All electrodes were charged to 4.0 V vs Na/Na^+^ with 1 m NaPF_6_ in PC + 5 wt % FEC. To ensure comparability, the capacity of the electrode material in each ARC tube was standardized to ∼2.5 mAh. Figure 4f shows the SHR versus temperature of NFMC and Ti-NFMC at 4.0 V vs Na/Na^+^. Minor exothermic activities between 50 and 200 °C were observed for both samples. The SHR increased significantly over 0.2 °C/min for desodiated NFMC and Ti-NFMC at ∼200 °C and ∼215 °C, respectively, suggesting the Ti^4+^ doping could increase the onset temperatures.

## 4 Conclusions

In this work, we demonstrate that we can perform an all-dry doping strategy to modify sodium-ion-layered oxide materials by processing surface coating on the hydroxide precursor before sintering. Dry particle fusion was used to coat TiO_2_ on the Ni_0.33_Fe_0.28_Cu_0.06_Mn_0.33_(OH)_2_ precursor and showed that core particles did not break after the mechanofusion process. The TiO_2_-coated Ni_0.33_Fe_0.28_Cu_0.06_Mn_0.33_(OH)_2_ sample was heated with NaOH in the air to form Ti-NFMC and compared with the Ni_0.33_Fe_0.28_Cu_0.06_Mn_0.33_(OH)_2_ sample without TiO_2_ coating with NaOH to form NFMC.

Postheat treatment analysis through XRD and ND revealed that all samples exhibited a well-developed single-phase layered structure, which suggests the evolution of Ti^4+^ as doping instead of maintaining surface coating anymore. This doping resulted in a slight increase in the lattice parameters. TEM coupled with EDS at both surface and bulk region of observing the Ti element confirmed that Ti had diffused uniformly inside the particles for the TiO_2_-coated material. XANES was used to confirm the decrease in Ni valence when doped with Ti^4+^ to maintain the charge balance in the pristine materials. Further, ^23^Na MAS NMR provided evidence of increased disorder in the transition metal arrangement around Na^+^ ions, suggesting Ti^4+^ integration into the local structure. In addition to understanding pristine materials, charge compensation mechanisms and thermal stability were also elucidated to the effect of Ti^4+^ doping on charged materials. In both Ti-NFMC and NFMC, XANES indicated that nickel primarily facilitated charge compensation across various states of charge, while iron contributed only at higher voltages (i.e., 4.0 V), with manganese and copper remaining redox-inactive. As the primary redox agent, nickel exhibits greater uniformity at high voltages when observed via TXM. Additionally, ARC tests demonstrated that charged Ti-NFMC exhibited enhanced thermal stability when reacting with electrolytes at elevated temperatures compared to NFMC. Electrochemically, Ti-NFMC showed better rate capability and stability in air. When paired with a hard carbon anode in single-layer pouch cells, Ti-NFMC/HC configurations maintained >85% of their initial capacity after 650 cycles at 25 °C, demonstrating significant improvements in cycling stability.

The application of dry particle fusion coating of TiO_2_ (and potentially other materials) onto a hydroxide precursor, followed by heat treatment in the presence of a sodium source, seems to be a superior method for producing advanced layered oxide cathodes for SIBs. This technique bypasses the need for direct air exposure during surface processing of sodium-ion cathode materials. The mechanofusion dry technique can be of exceptional quality, capable of enhancing the cycle life of various materials used in SIBs. It shows great promise for the future of SIB material processing, offering the potential for batteries with a long lifetime through an approach that is cost-efficient, solvent-free, scalable, and environmentally benign.

## Supplementary Material



## Notes

Further scanning electron microscopy, X-ray diffraction, neutron diffraction, solid-state nuclear magnetic resonance, extended X-ray absorption fine structure, and electrochemical data (PDF)
