# Supplementary material for: Advancing Sodium-Ion Battery Cathodes: A Low-Cost, Eco-Friendly Mechanofusion Route from TiO2 Coating to Ti4+ Doping
Source: Chem Mater. 2025 Aug 1;37(15):6059–68. doi: 10.1021/acs.chemmater.5c01485 (PMC12355685; doi:10.1021/acs.chemmater.5c01485)
Supplement: Supplementary file 1 [file cm5c01485_si_001.pdf]

# Supporting Information

## Advancing Sodium-Ion Battery Cathodes: A Low-Cost, Eco-Friendly Mechanofusion Route from $TiO_2$ Coating to $Ti^{4+}$ Doping

Vadim Shipitsyn[a,b§], Guanyi Wang[c§], Wenhua Zuo[d*], Ning Zhang[e], Yongkang Jin[f], Kangxuan Xia[g], Cheng Li[h], Rishivandhiga Jayakumar[a,b], Chanmonirath (Michael) Chak[a,b], Yan-Yan Hu[f], Riqiang Fu[i], Guiliang Xu[d], Xianghui Xiao[j], Jialin Mao[k], Wenbin Yin[k], Enyuan Hu[g], Eric McCalla[l], Lin Ma[a,b,m*]

a. Department of Mechanical Engineering and Engineering Science, The University of North Carolina at Charlotte, Charlotte, NC 28223, USA
b. Battery Complexity, Autonomous Vehicle and Electrification (BATT CAVE) Research Center, The University of North Carolina at Charlotte, Charlotte, NC 28223, USA
c. Applied Materials Division, Argonne National Laboratory, 9700 S Cass Ave, Lemont, IL 60439 USA
d. Chemical Sciences and Engineering Division, Argonne National Laboratory, 9700 S Cass Ave, Lemont, IL 60439 USA
e. Department of Physics and Atmospheric Science, Dalhousie University, Halifax B3H 4R2, Canada
f. Department of Chemistry and Biochemistry, Florida State University, Tallahassee, FL, 32306 USA
g. Chemistry Division, Brookhaven National Laboratory, Upton, New York 11973, USA
h. Neutron Scattering Division, Oak Ridge National Laboratory (ORNL), Oak Ridge, Tennessee 37831, USA
i. The National High Magnetic Field Laboratory, Florida State University, Tallahassee, FL 32310, USA
j. National Synchrotron Light Source II, Brookhaven National Laboratory, Upton, NY 11973, USA
k. Celgard, LLC, Concord, NC 28027, USA
l. Department of Chemistry, McGill University, Montreal, Quebec H3A 0B8, Canada
m. Department of Applied Physical Sciences, University of North Carolina, Chapel Hill, NC 27514, USA

E-mail: wzuo@anl.gov; l.ma@charlotte.edu; l.ma@unc.edu
§ these authors contributed equally

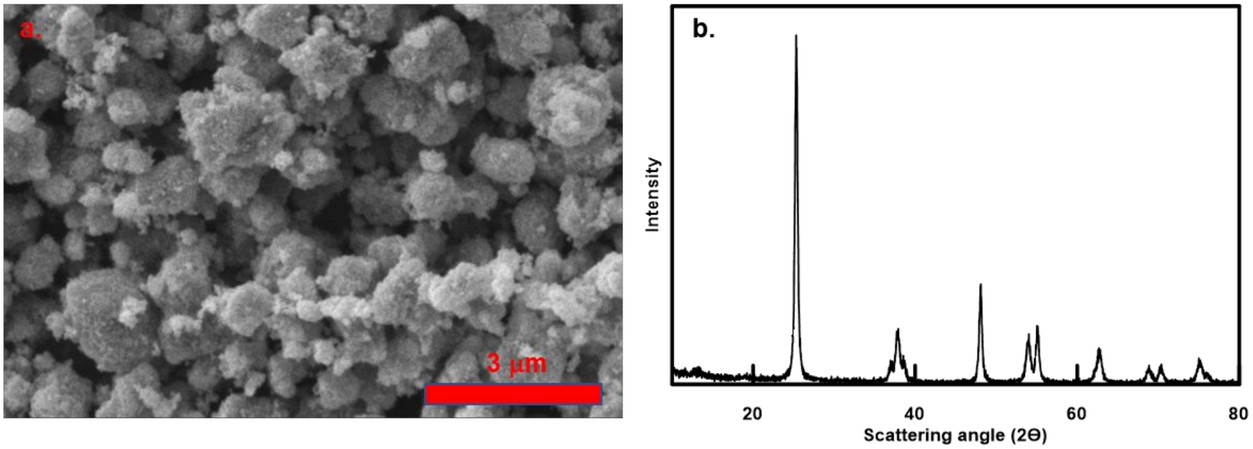


**Figure S1.** (a) SEM and (b) XRD of $TiO_2$ used as surface coating materials.

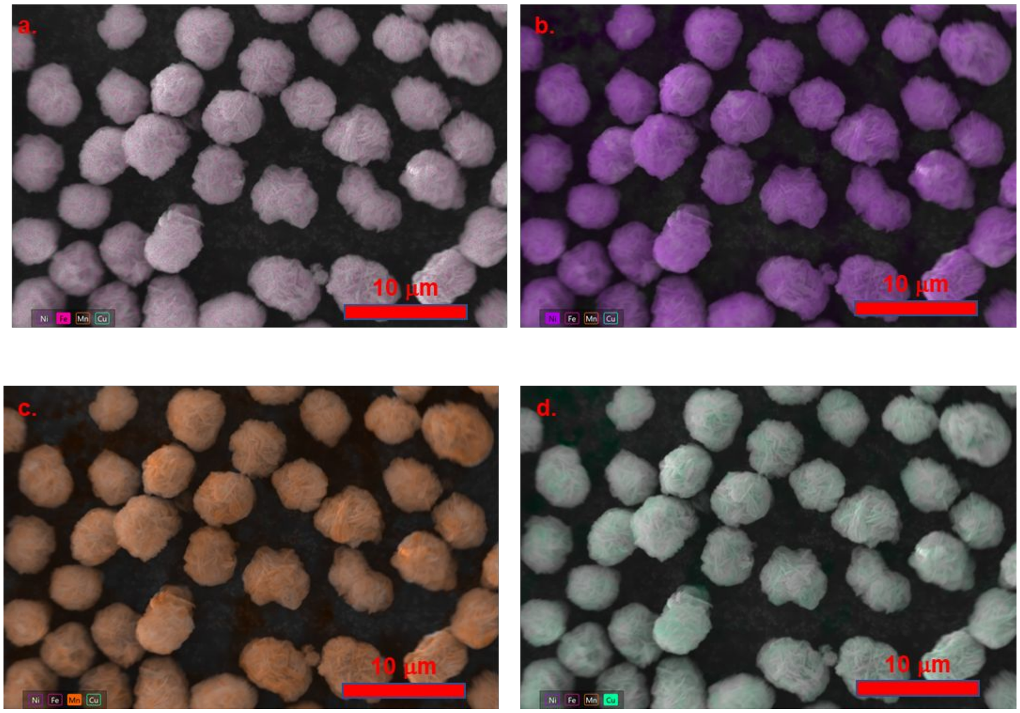


**Figure S2.** SEM images of $Ni_{0.33}Fe_{0.28}Cu_{0.06}Mn_{0.33}(OH)_2$ precursor with EDX on (a) Fe, (b) Ni, (c) Mn, and (d) Cu.

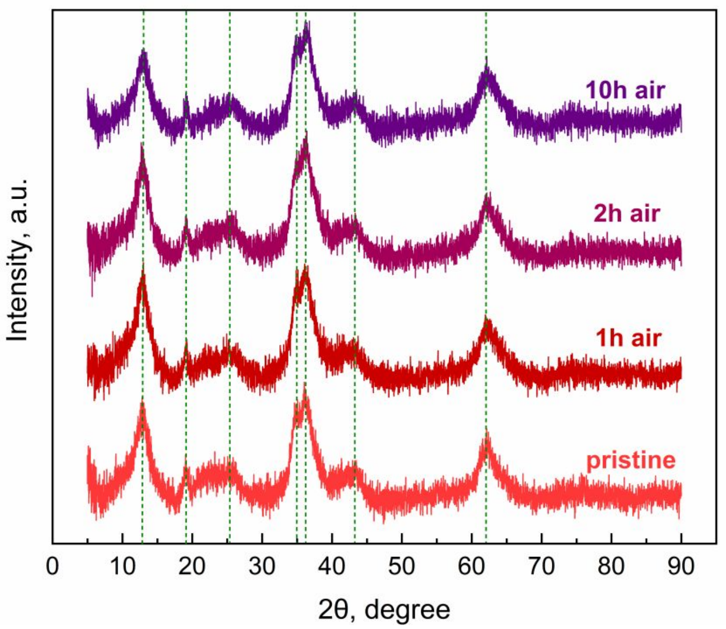


**Figure S3.** XRD of $Ni_{0.33}Fe_{0.28}Cu_{0.06}Mn_{0.33}(OH)_2$ precursor with air exposure for different time of period including (a) 0h, (b) 1h, (c) 2h, and (d) 10h at room temperature.

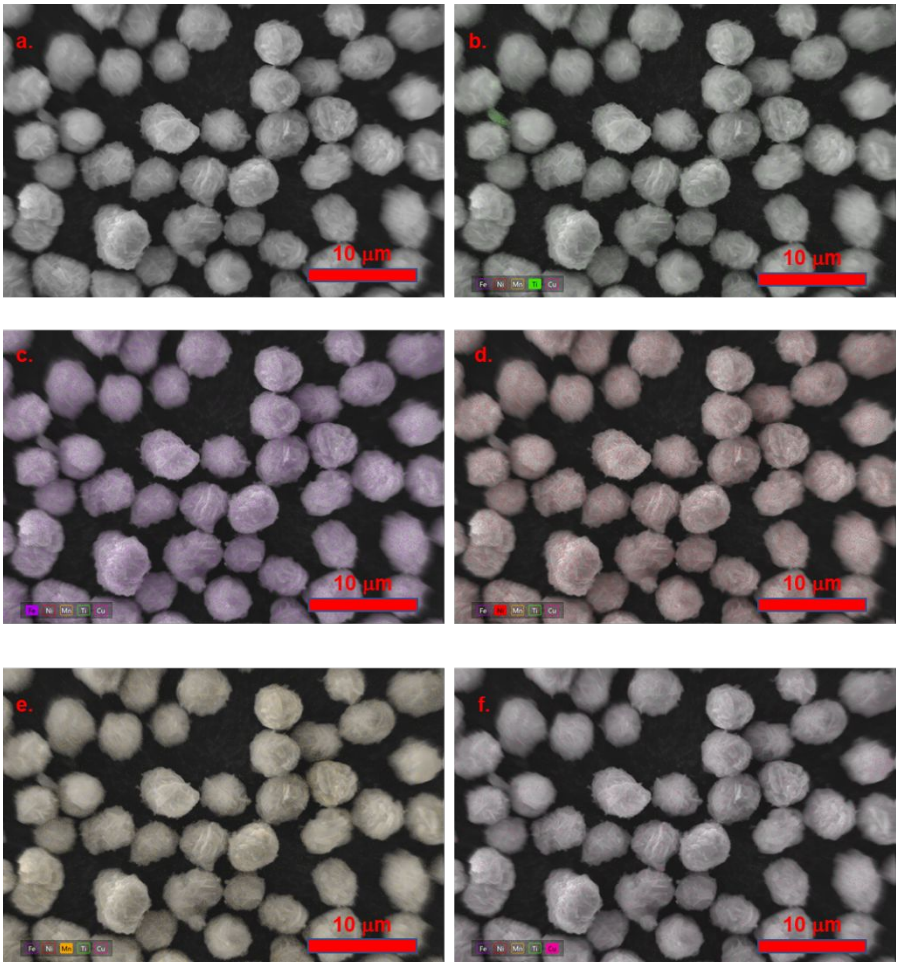


**Figure S4.** SEM images of (a) $Ni_{0.33}Fe_{0.28}Cu_{0.06}Mn_{0.33}(OH)_2$ precursor coated with 1 wt% $TiO_2$ with EDX on (b) Ti, (c) Fe, (d) Ni, (e) Mn, and (f) Cu.

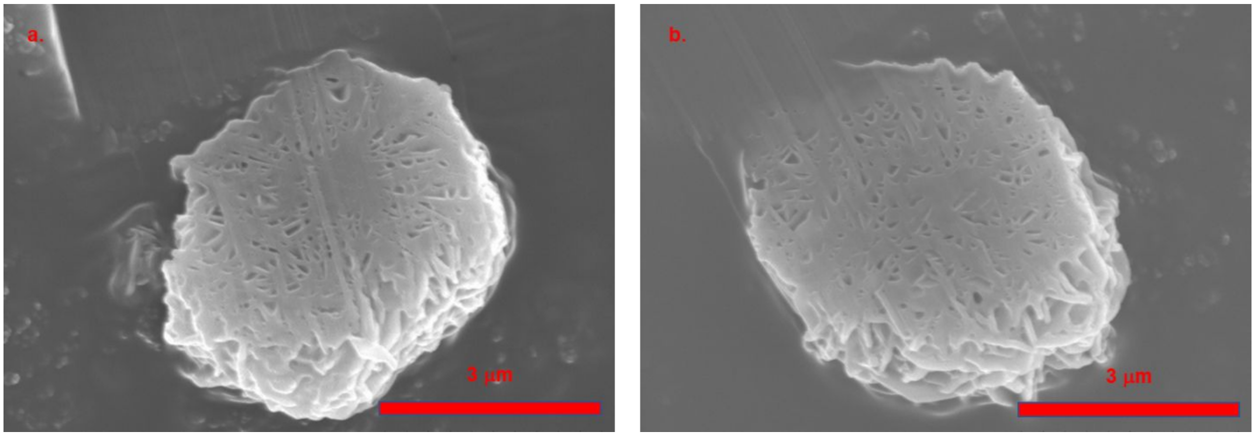


**Figure S5.** Cross-section SEM images of (a) $Ni_{0.33}Fe_{0.28}Cu_{0.06}Mn_{0.33}(OH)_2$ precursor and (b) $Ni_{0.33}Fe_{0.28}Cu_{0.06}Mn_{0.33}(OH)_2$ precursor coated with 1 wt.% $TiO_2$.

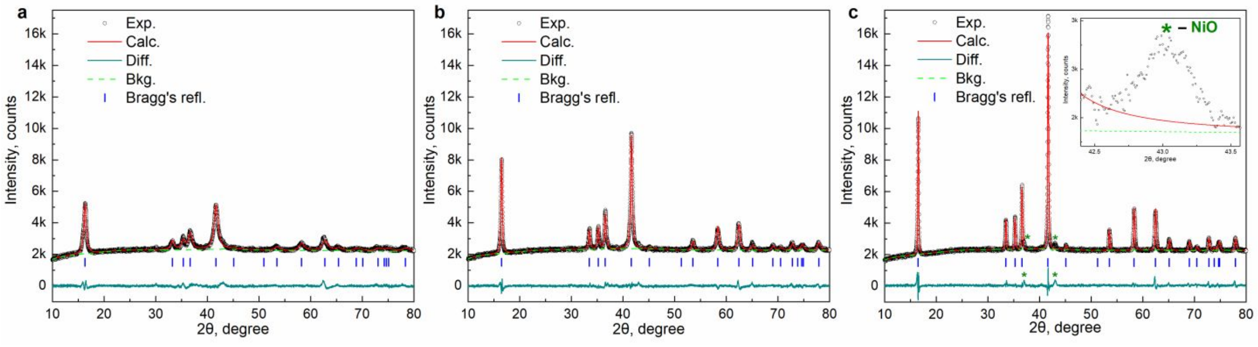


**Figure S6.** XRD of $NaNi_{0.33}Fe_{0.28}Mn_{0.33}Cu_{0.06}O_2$ (NFMC) sintered at (a) 650°C, (b) 750°C, and (c) 850°C for 15h in the air according to a molar ratio of 1:1 between precursor and NaOH.

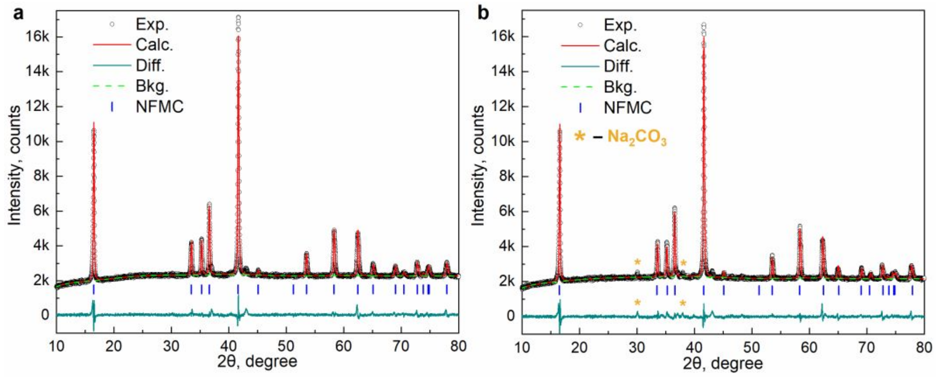


**Figure S7.** XRD of $NaNi_{0.33}Fe_{0.28}Mn_{0.33}Cu_{0.06}O_2$ (NFMC) sintered with different molar ratio between precursor and NaOH including (a) 1:1, (b) 1:1.3 at 850°C for 15h in the air.

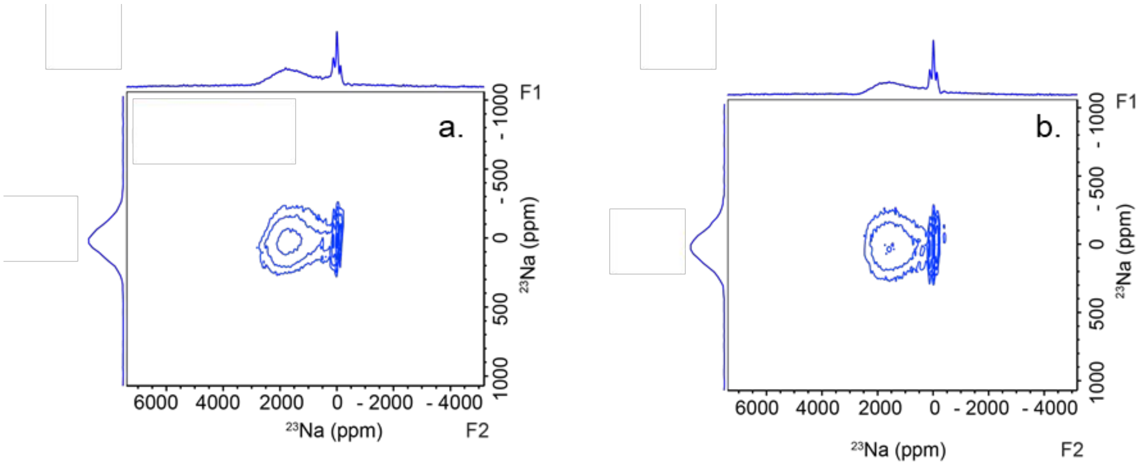


**Figure S8.** $^{23}$Na MAS 2D NMR spectrum for (a) NFMC and (b) Ti-NFMC.

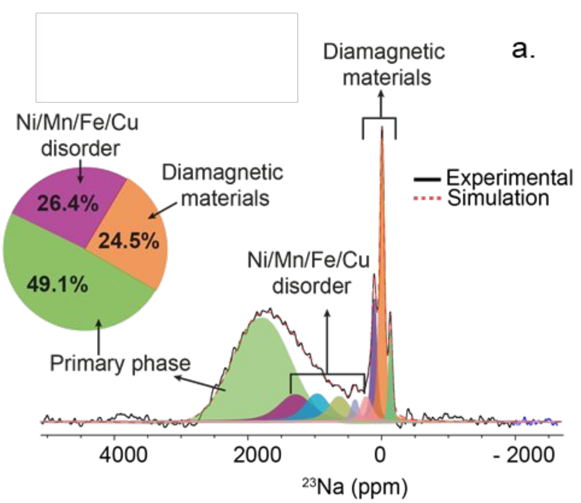


**Figure S9.** Deconvolution of $^{23}$Na MAS NMR spectrum for (a) NFMC and (b) Ti-NFMC. The projected spectrum from the sheared data contains only pure isotropic peaks. Each peak corresponds to a distinct Na local environment, allowing for accurate site identification and quantification.

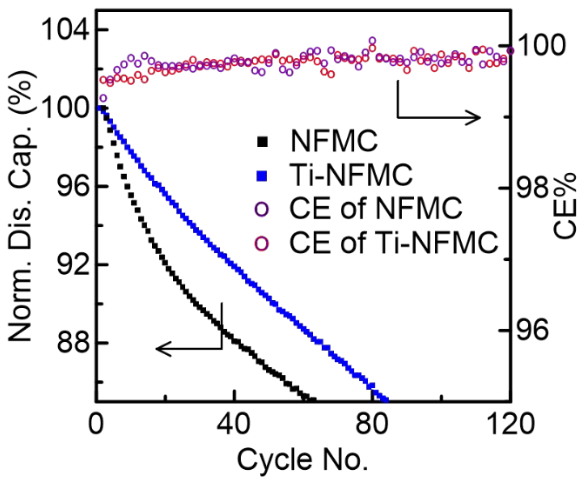


**Figure S10.** Cycling stability of NFMC and Ti-NFMC cathodes coupled with Na metal anode in coin cells in the voltage range 2.0–4.0 V at 0.5 C and 25°C with 1m $NaPF_6$ in PC + 5wt.% FEC.

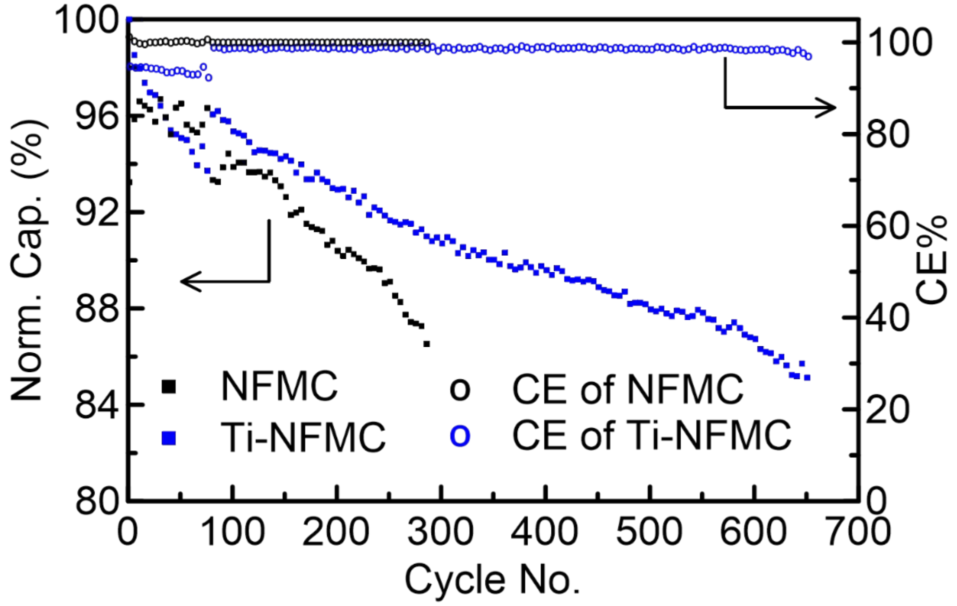


**Figure S11.** Cycling stability of NFMC and Ti-NFMC cathodes, coupled with hard carbon anode in single layer pouch cells, in the voltage range 1.5–4.0 V at C/3 and 25°C with 1m $NaPF_6$ in PC + 2 wt.% DTD.

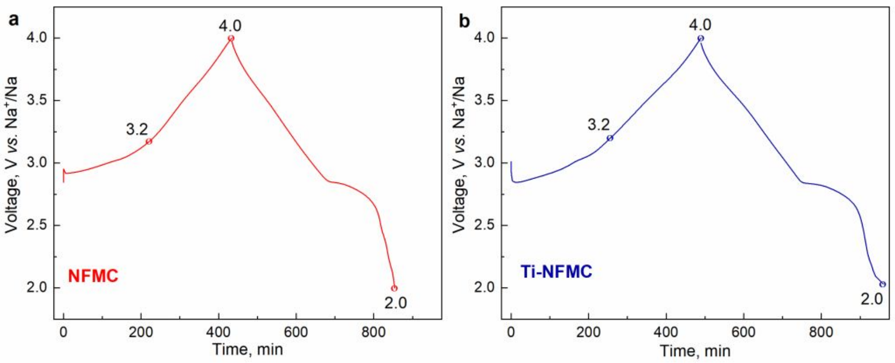


**Figure S12.** Voltage vs. time for (a) NFMC and (b) Ti-NFMC during first cycle charge and discharge using CC–CV (C/10 – C/100) at 40°C. Samples were taken at 3.2V, 4.0V and 2.0V for XAS characterizations.

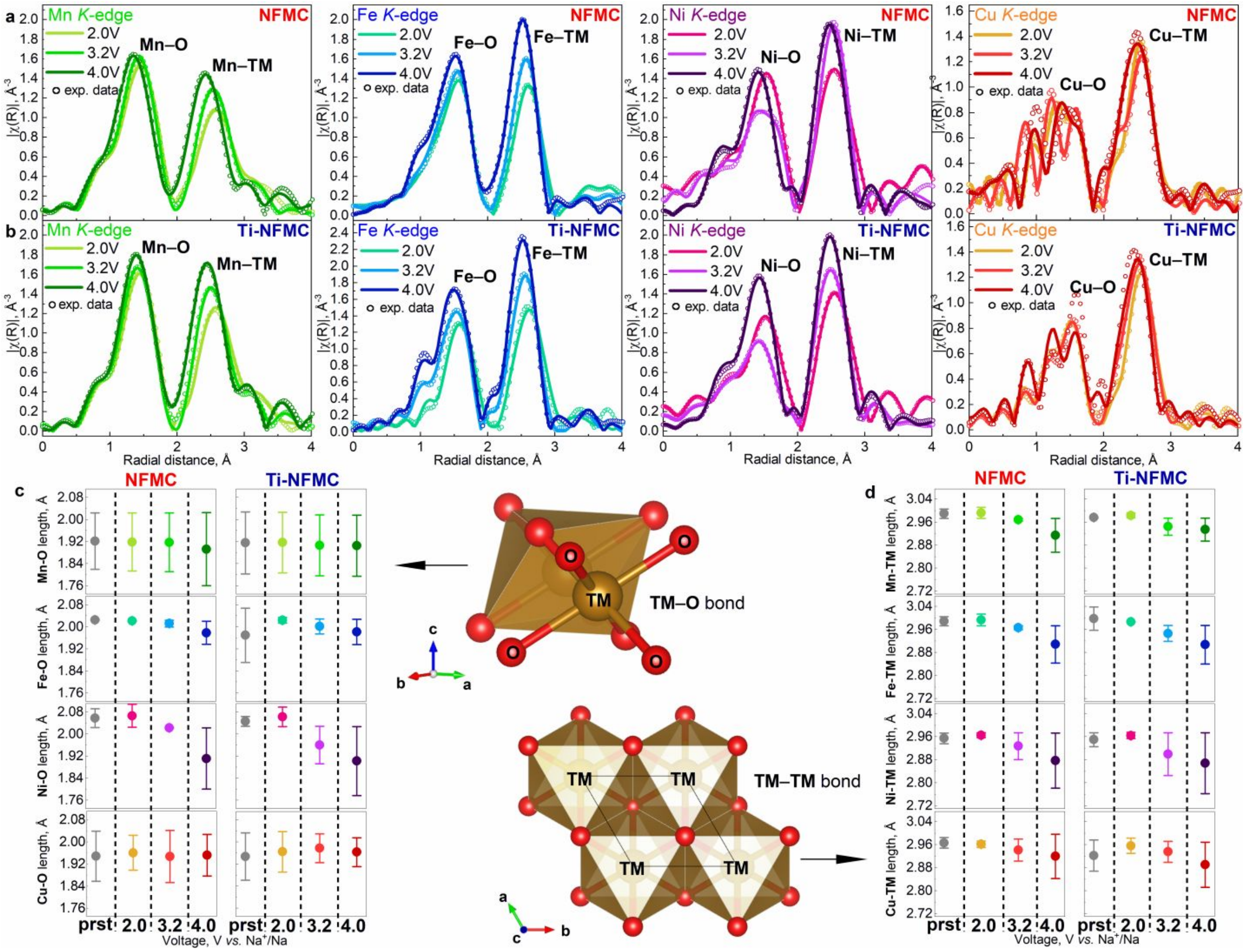


**Figure S13.** Fourier transform magnitudes of $K^3$-weighted χ(k) at Mn K-edge, Fe K-edge, Ni K-edge, and Cu K-edge for (a) NFMC and (b) Ti-NFMC with (c) TM–O and (d) TM–TM bond lengths.

**Supplementary Table 1.** Element analysis of the cathodes using ICP-OES. Atomic ratios were normalized to Ni + Fe + Mn + Cu + Ti = 1.0. The overall transition metal composition aligns well with the expectation.

| | Ti | Ni | Fe | Mn | Cu |
|---|---|---|---|---|---|
| NFMC | N/A | 0.337 | 0.275 | 0.329 | 0.059 |
| | N/A | 0.329 | 0.283 | 0.333 | 0.055 |
| | N/A | 0.334 | 0.279 | 0.331 | 0.056 |
| Ti-NFMC | 0.015 | 0.328 | 0.279 | 0.325 | 0.053 |
| | 0.011 | 0.325 | 0.281 | 0.327 | 0.056 |
| | 0.013 | 0.324 | 0.279 | 0.323 | 0.061 |

**Supplementary Table 2.** A summary of XRD refinement parameters of (a) NFMC and (b) Ti-NFMC.

**a.**

| NFMC | | | | | | |
|---|---|---|---|---|---|---|
| Space group: $R\overline{3}m$ | | | | | | |
| $a$ = 2.97174(6) Å; $c$ = 15.97364(27) Å; $V$ = 122.1678(24) Å$^3$ | | | | | | |
| $R_w$ = 2.37%; $R_F$ = 2.68%; GOF = 1.31 | | | | | | |
| Atom | Site | x/a | y/b | z/c | Occupancy | $U_{iso}$ (Å$^2$) |
| Na | 3b | 0.0000 | 0.0000 | 0.5000 | 1.0000 | 0.0192 |
| Ni | 3a | 0.0000 | 0.0000 | 0.0000 | 0.3300 | 0.0015 |
| Fe | 3a | 0.0000 | 0.0000 | 0.0000 | 0.2800 | 0.0072 |
| Mn | 3a | 0.0000 | 0.0000 | 0.0000 | 0.3300 | 0.0103 |
| Cu | 3a | 0.0000 | 0.0000 | 0.0000 | 0.0600 | 0.0276 |
| O | 6c | 0.0000 | 0.0000 | 0.2675 | 1.0000 | 0.0273 |

**b.**

| Ti-NFMC | | | | | | |
|---|---|---|---|---|---|---|
| Space group: $R\overline{3}m$ | | | | | | |
| $a$ = 2.97493(6) Å; $c$ = 15.98001(29) Å; $V$ = 122.4785(24) Å$^3$ | | | | | | |
| $R_w$ = 2.27%; $R_F$ = 2.27%; GOF = 1.24 | | | | | | |
| Atom | Site | x/a | y/b | z/c | Occupancy | $U_{iso}$ (Å$^2$) |
| Na | 3b | 0.0000 | 0.0000 | 0.5000 | 1.0000 | 0.0181 |
| Ni | 3a | 0.0000 | 0.0000 | 0.0000 | 0.3260 | 0.0014 |
| Fe | 3a | 0.0000 | 0.0000 | 0.0000 | 0.2770 | 0.0071 |
| Mn | 3a | 0.0000 | 0.0000 | 0.0000 | 0.3260 | 0.0109 |
| Cu | 3a | 0.0000 | 0.0000 | 0.0000 | 0.0590 | 0.0038 |
| Ti | 3a | 0.0000 | 0.0000 | 0.0000 | 0.0120 | 0.3260 |
| O | 6c | 0.0000 | 0.0000 | 0.2692 | 1.0000 | 0.0210 |

**Supplementary Table 3.** A summary of ND refinement parameters of (a) NFMC and (b) Ti-NFMC.

**a.**

| NFMC<br>Space group: $R\overline{3}m$<br>$a$ = 2.9744(1) Å; $c$ = 15.9998(6) Å; $V$ = 122.589 Å$^3$<br>$R_w$ = 8.77%; $R_F$ = 6.23%; GOF = 3.38 | | | | | |
|---|---|---|---|---|---|
| Atom | Site | x/a | y/b | z/c | $U_{iso}$ (Å$^2$) |
| Na | 3b | 0.0000 | 0.0000 | 0.5000 | 0.01 |
| Ni | 3a | 0.0000 | 0.0000 | 0.0000 | 0.005 |
| Fe | 3a | 0.0000 | 0.0000 | 0.0000 | 0.005 |
| Mn | 3a | 0.0000 | 0.0000 | 0.0000 | 0.01 |
| Cu | 3a | 0.0000 | 0.0000 | 0.0000 | 0.01 |
| O | 6c | 0.0000 | 0.0000 | 0.2678 | 0.01 |

**b.**

| Ti-NFMC<br>Space group: $R\overline{3}m$<br>$a$ = 2.9751(5) Å; $c$ = 16.0079(4) Å; $V$ = 122.711 Å$^3$<br>$R_w$ = 8.78%; $R_F$ = 6.36%; GOF = 3.35 | | | | | |
|---|---|---|---|---|---|
| Atom | Site | x/a | y/b | z/c | $U_{iso}$ (Å$^2$) |
| Na | 3b | 0.0000 | 0.0000 | 0.5000 | 0.01 |
| Ni | 3a | 0.0000 | 0.0000 | 0.0000 | 0.005 |
| Fe | 3a | 0.0000 | 0.0000 | 0.0000 | 0.005 |
| Mn | 3a | 0.0000 | 0.0000 | 0.0000 | 0.01 |
| Cu | 3a | 0.0000 | 0.0000 | 0.0000 | 0.01 |
| Ti | 3a | 0.0000 | 0.0000 | 0.0000 | 0.01 |
| O | 6c | 0.0000 | 0.0000 | 0.2679 | 0.01 |

**Supplementary Table 4.** A summary of EXAFS results of (a) NFMC and (b) Ti-NFMC.

**a-1.** The best fit parameters for the *ex situ* NFMC **pristine**
$\Delta E_0$ =0.13 +/- 5.26, $S_0^2$ = 0.851 +/- 0.117, R-factor = 0.0019

| Bond | N | R, Å | Sigma squared $\sigma^2$, $10^{-3}$ Å$^2$ |
|---|---|---|---|
| Fe-O | 6 | 2.024 | 6.17 |
| Fe-TM | 6 | 2.988 | 6.49 |
| Fe-Na | 6 | 3.184 | 14.6 |

**a-2.** The best fit parameters for the *ex situ* NFMC **charged to 3.2 V** vs. $Na^+$/Na
$\Delta E_0$ =0.00018 +/- 6.71789, $S_0^2$ = 0.889 +/- 0.008, R-factor = 0.0075

| Bond | N | R, Å | Sigma squared $\sigma^2$, $10^{-3}$ Å$^2$ |
|---|---|---|---|
| Fe-O | 6 | 2.011 | 6.65 |
| Fe-TM | 6 | 2.966 | 6.80 |
| Fe-Na | 6 | 3.161 | 17.9 |

**a-3.** The best fit parameters for the *ex situ* NFMC **charged to 4.0 V** vs. $Na^+$/Na
$\Delta E_0$ = 3.54 +/- 1.27, $S_0^2$ = 0.927 +/- 0.096, R-factor = 0.0018

| Bond | N | R, Å | Sigma squared $\sigma^2$, $10^{-3}$ Å$^2$ |
|---|---|---|---|
| Fe-O | 6 | 1.978 | 7.70 |
| Fe-TM | 6 | 2.907 | 6.39 |
| Fe-Na | 6 | 3.098 | 17.0 |

**a-4.** The best fit parameters for the *ex situ* NFMC **discharged to 2.0 V** vs. $Na^+$/Na
$\Delta E_0$ = 5.86 +/- 5.65, $S_0^2$ = 0.807 +/- 0.111, R-factor = 0.0034

| Bond | N | R, Å | Sigma squared $\sigma^2$, $10^{-3}$ Å$^2$ |
|---|---|---|---|
| Fe-O | 6 | 2.021 | 6.09 |
| Fe-TM | 6 | 2.993 | 6.79 |
| Fe-Na | 6 | 3.190 | 14.2 |

**a-5.** The best fit parameters for the *ex situ* NFMC **pristine**
$\Delta E_0$ = 8.22 +/- 5.33, $S_0^2$ = 0.828 +/- 0.362, R-factor = 0.0066

| Bond | N | R, Å | Sigma squared $\sigma^2$, $10^{-3}$ Å$^2$ |
|---|---|---|---|
| Ni-O | 6 | 2.024 | 6.17 |
| Ni-TM | 6 | 2.988 | 6.49 |
| Ni-Na | 6 | 3.184 | 14.6 |

**a-6.** The best fit parameters for the *ex situ* NFMC **charged to 3.2 V** vs. $Na^+$/Na
$\Delta E_0$ =0.00018 +/- 6.718, $S_0^2$ = 0.889 +/- 0.008, R-factor = 0.0075

| Bond | N | R, Å | Sigma squared $\sigma^2$, $10^{-3}$ Å$^2$ |
|---|---|---|---|
| Ni-O | 6 | 2.011 | 6.65 |
| Ni-TM | 6 | 2.966 | 6.80 |
| Ni-Na | 6 | 3.161 | 17.9 |

**a-7.** The best fit parameters for the *ex situ* NFMC **charged to 4.0 V** vs. $Na^+$/Na
$\Delta E_0$ = 8.282 +/- 4.085, $S_0^2$ = 0.707 +/- 0.724, R-factor = 0.0051

| Bond | N | R, Å | Sigma squared $\sigma^2$, $10^{-3}$ Å$^2$ |
|---|---|---|---|
| Ni-O | 6 | 1.911 | 7.92 |
| Ni-TM | 6 | 2.877 | 4.04 |
| Ni-Na | 6 | 3.066 | 50.6 |

**a-8.** The best fit parameters for the *ex situ* NFMC **discharged to 2.0 V** vs. $Na^+$/Na
$\Delta E_0$ = 4.70 +/- 6.57, $S_0^2$ = 0.836 +/- 0.796, R-factor = 0.0034

| Bond | N | R, Å | Sigma squared $\sigma^2$, $10^{-3}$ Å$^2$ |
|---|---|---|---|
| Ni-O | 6 | 2.066 | 5.53 |
| Ni-TM | 6 | 2.965 | 5.64 |
| Ni-Na | 6 | 3.160 | 30.7 |

**a-9.** The best fit parameters for the *ex situ* NFMC **pristine**
$\Delta E_0$ = 4.59 +/- 1.88, $S_0^2$ = 0.936 +/- 0.353, R-factor = 0.0042

| Bond | N | R, Å | Sigma squared $\sigma^2$, $10^{-3}$ Å$^2$ |
|---|---|---|---|
| Mn-O | 6 | 1.933 | 3.96 |
| Mn-TM | 6 | 2.992 | 4.41 |
| Mn-Na | 6 | 3.188 | 28.8 |

**a-10.** The best fit parameters for the *ex situ* NFMC **charged to 3.2 V** vs. $Na^+$/Na
$\Delta E_0$ = 7.19 +/- 1.99, $S_0^2$ = 0.919 +/- 0.244, R-factor = 0.0021

| Bond | N | R, Å | Sigma squared $\sigma^2$, $10^{-3}$ Å$^2$ |
|---|---|---|---|
| Mn-O | 6 | 1.915 | 3.80 |
| Mn-TM | 6 | 2.968 | 7.01 |
| Mn-Na | 6 | 3.163 | 17.3 |

**a-11.** The best fit parameters for the *ex situ* NFMC **charged to 4.0 V** vs. $Na^+$/Na
$\Delta E_0$ = 8.47 +/- 1.61, $S_0^2$ = 0.546 +/- 0.078, R-factor = 0.0069

| Bond | N | R, Å | Sigma squared $\sigma^2$, $10^{-3}$ Å$^2$ |
|---|---|---|---|
| Mn-O | 6 | 1.885 | 0.21 |
| Mn-TM | 6 | 2.906 | 0.95 |
| Mn-Na | 6 | 3.097 | 8.35 |

**a-12.** The best fit parameters for the *ex situ* NFMC **discharged to 2.0 V** vs. $Na^+$/Na
$\Delta E_0$ = 4.79 +/- 2.45, $S_0^2$ = 1.126 +/- 0.554, R-factor = 0.0067

| Bond | N | R, Å | Sigma squared $\sigma^2$, $10^{-3}$ Å$^2$ |
|---|---|---|---|
| Mn-O | 6 | 1.935 | 4.87 |
| Mn-TM | 6 | 2.999 | 4.99 |
| Mn-Na | 6 | 3.196 | 33.1 |

**a-13.** The best fit parameters for the *ex situ* NFMC **pristine**
$\Delta E_0$ = 7.89 +/- 3.29, $S_0^2$ = 1.198 +/- 0.462, R-factor = 0.0271

| Bond | N | R, Å | Sigma squared $\sigma^2$, $10^{-3}$ Å$^2$ |
|---|---|---|---|
| Cu-O | 6 | 1.948 | 12.6 |
| Cu-TM | 6 | 2.966 | 10.9 |
| Cu-Na | 6 | 3.161 | 180.1 |

**a-14.** The best fit parameters for the *ex situ* NFMC **charged to 3.2 V** vs. $Na^+$/Na
$\Delta E_0$ = 8.13 +/- 2.16, $S_0^2$ = 1.177 +/- 0.511, R-factor = 0.0147

| Bond | N | R, Å | Sigma squared $\sigma^2$, $10^{-3}$ Å$^2$ |
|---|---|---|---|
| Cu-O | 6 | 1.947 | 11.9 |
| Cu-TM | 6 | 2.941 | 12.2 |
| Cu-Na | 6 | 3.135 | 112.4 |

**a-15.** The best fit parameters for the *ex situ* NFMC **charged to 4.0 V** vs. $Na^+$/Na
$\Delta E_0$ = 6.07 +/- 3.13, $S_0^2$ = 1.040 +/- 0.972, R-factor = 0.0683

| Bond | N | R, Å | Sigma squared $\sigma^2$, $10^{-3}$ Å$^2$ |
|---|---|---|---|
| Cu-O | 6 | 1.951 | 8.24 |
| Cu-TM | 6 | 2.919 | 10.5 |
| Cu-Na | 6 | 3.111 | 63.8 |

**a-16.** The best fit parameters for the *ex situ* NFMC **discharged to 2.0 V** vs. $Na^+$/Na
$\Delta E_0$ = 7.87 +/- 1.77, $S_0^2$ = 0.839 +/- 0.294, R-factor = 0.0093

| Bond | N | R, Å | Sigma squared $\sigma^2$, $10^{-3}$ Å$^2$ |
|---|---|---|---|
| Cu-O | 6 | 1.960 | 12.2 |
| Cu-TM | 6 | 2.961 | 8.04 |
| Cu-Na | 6 | 3.156 | 80.7 |

**b-1.** The best fit parameters for the *ex situ* Ti-NFMC **pristine**
$\Delta E_0$ = 16.89 +/- 3.25, $S_0^2$ = 1.029 +/- 0.410, R-factor = 0.0975

| Bond | N | R, Å | Sigma squared $\sigma^2$, $10^{-3}$ Å$^2$ |
|---|---|---|---|
| Fe-O | 6 | 1.969 | 0.63 |
| Fe-TM | 6 | 2.997 | 17.7 |
| Fe-Na | 6 | 3.191 | 0.75 |

**b-2.** The best fit parameters for the *ex situ* Ti-NFMC **charged to 3.2 V** vs. $Na^+$/Na
$\Delta E_0$ = 14.54 +/- 6.08, $S_0^2$ = 0.680 +/- 0.763, R-factor = 0.0074

| Bond | N | R, Å | Sigma squared $\sigma^2$, $10^{-3}$ Å$^2$ |
|---|---|---|---|
| Fe-O | 6 | 2.001 | 3.93 |
| Fe-TM | 6 | 2.946 | 4.03 |
| Fe-Na | 6 | 3.137 | 31.97 |

**b-3.** The best fit parameters for the *ex situ* Ti-NFMC **charged to 4.0 V** vs. $Na^+$/Na
$\Delta E_0$ = 3.549 +/- 1.699, $S_0^2$ = 0.791 +/- 0.219, R-factor = 0.0059

| Bond | N | R, Å | Sigma squared $\sigma^2$, $10^{-3}$ Å$^2$ |
|---|---|---|---|
| Fe-O | 6 | 1.981 | 5.53 |
| Fe-TM | 6 | 2.907 | 4.37 |
| Fe-Na | 6 | 3.096 | 49.3 |

**b-4.** The best fit parameters for the *ex situ* Ti-NFMC **discharged to 2.0 V** vs. $Na^+$/Na
$\Delta E_0$ = 5.97 +/- 4.95, $S_0^2$ = 0.923 +/- 0.405, R-factor = 0.0311

| Bond | N | R, Å | Sigma squared $\sigma^2$, $10^{-3}$ Å$^2$ |
|---|---|---|---|
| Fe-O | 6 | 2.023 | 4.79 |
| Fe-TM | 6 | 2.986 | 6.80 |
| Fe-Na | 6 | 3.180 | 14.7 |

**b-5.** The best fit parameters for the *ex situ* Ti-NFMC **pristine**
$\Delta E_0$ = 9.42 +/- 6.80, $S_0^2$ = 0.686 +/- 0.362, R-factor = 0.0010

| Bond | N | R, Å | Sigma squared $\sigma^2$, $10^{-3}$ Å$^2$ |
|---|---|---|---|
| Ni-O | 6 | 2.045 | 5.41 |
| Ni-TM | 6 | 2.949 | 3.81 |
| Ni-Na | 6 | 3.141 | 57.4 |

**b-6.** The best fit parameters for the *ex situ* Ti-NFMC **charged to 3.2 V** vs. $Na^+$/Na
$\Delta E_0$ = 65.40 +/- 11.59, $S_0^2$ = 0.889 +/- 2.119, R-factor = 0.0033

| Bond | N | R, Å | Sigma squared $\sigma^2$, $10^{-3}$ Å$^2$ |
|---|---|---|---|
| Ni-O | 6 | 1.959 | 13.9 |
| Ni-TM | 6 | 2.899 | 6.48 |
| Ni-Na | 6 | 3.088 | 24.3 |

**b-7.** The best fit parameters for the *ex situ* Ti-NFMC **charged to 4.0 V** vs. $Na^+$/Na
$\Delta E_0$ = 12.14 +/- 4.38, $S_0^2$ = 0.650 +/- 0.635, R-factor = 0.0029

| Bond | N | R, Å | Sigma squared $\sigma^2$, $10^{-3}$ Å$^2$ |
|---|---|---|---|
| Ni-O | 6 | 1.902 | 5.49 |
| Ni-TM | 6 | 2.868 | 2.95 |
| Ni-Na | 6 | 3.054 | 53.6 |

**b-8.** The best fit parameters for the *ex situ* Ti-NFMC **discharged to 2.0 V** vs. $Na^+$/Na
$\Delta E_0$ = 4.31 +/- 5.65, $S_0^2$ = 0.766 +/- 0.465, R-factor = 0.0012

| Bond | N | R, Å | Sigma squared $\sigma^2$, $10^{-3}$ Å$^2$ |
|---|---|---|---|
| Ni-O | 6 | 2.062 | 7.46 |
| Ni-TM | 6 | 2.964 | 5.44 |
| Ni-Na | 6 | 3.156 | 37.79 |

**b-9.** The best fit parameters for the *ex situ* Ti-NFMC **pristine**
$\Delta E_0$ = 6.32 +/- 6.66, $S_0^2$ = 0.587 +/- 0.260, R-factor = 0.0069

| Bond | N | R, Å | Sigma squared $\sigma^2$, $10^{-3}$ Å$^2$ |
|---|---|---|---|
| Mn-O | 6 | 1.915 | 0.41 |
| Mn-TM | 6 | 2.976 | 2.10 |
| Mn-Na | 6 | 3.169 | 14.9 |

**b-10.** The best fit parameters for the *ex situ* Ti-NFMC **charged to 3.2 V** vs. $Na^+$/Na
$\Delta E_0$ = 16.83 +/- 5.92, $S_0^2$ = 0.546 +/- 0.455, R-factor = 0.0052

| Bond | N | R, Å | Sigma squared $\sigma^2$, $10^{-3}$ Å$^2$ |
|---|---|---|---|
| Mn-O | 6 | 1.906 | 0.64 |
| Mn-TM | 6 | 2.944 | 2.05 |
| Mn-Na | 6 | 3.135 | 30.2 |

**b-11.** The best fit parameters for the *ex situ* Ti-NFMC **charged to 4.0 V** vs. $Na^+$/Na
$\Delta E_0$ = 7.98 +/- 3.91, $S_0^2$ = 0.464 +/- 0.359, R-factor = 0.0034

| Bond | N | R, Å | Sigma squared $\sigma^2$, $10^{-3}$ Å$^2$ |
|---|---|---|---|
| Mn-O | 6 | 1.905 | 2.82 |
| Mn-TM | 6 | 2.934 | 2.01 |
| Mn-Na | 6 | 3.124 | 23.2 |

**b-12.** The best fit parameters for the *ex situ* Ti-NFMC **discharged to 2.0 V** vs. $Na^+$/Na
$\Delta E_0$ = 3.56 +/- 5.90, $S_0^2$ = 0.513 +/- 0.109, R-factor = 0.0058

| Bond | N | R, Å | Sigma squared $\sigma^2$, $10^{-3}$ Å$^2$ |
|---|---|---|---|
| Mn-O | 6 | 1.916 | 0.31 |
| Mn-TM | 6 | 2.983 | 2.23 |
| Mn-Na | 6 | 3.177 | 15.03 |

**b-13.** The best fit parameters for the *ex situ* Ti-NFMC **pristine**
$\Delta E_0$ = 13.76 +/- 3.29, $S_0^2$ = 0.703 +/- 0.186, R-factor = 0.0419

| Bond | N | R, Å | Sigma squared $\sigma^2$, $10^{-3}$ Å$^2$ |
|---|---|---|---|
| Cu-O | 6 | 1.947 | 12.5 |
| Cu-TM | 6 | 2.922 | 7.77 |
| Cu-Na | 6 | 3.111 | 79.2 |

**b-14.** The best fit parameters for the *ex situ* Ti-NFMC **charged to 3.2 V** vs. $Na^+$/Na
$\Delta E_0$ = 6.47 +/- 3.63, $S_0^2$ = 1.297 +/- 0.340, R-factor = 0.0271

| Bond | N | R, Å | Sigma squared $\sigma^2$, $10^{-3}$ Å$^2$ |
|---|---|---|---|
| Cu-O | 6 | 1.977 | 10.2 |
| Cu-TM | 6 | 2.935 | 12.1 |
| Cu-Na | 6 | 3.126 | 126.9 |

**b-15.** The best fit parameters for the *ex situ* Ti-NFMC **charged to 4.0 V** vs. $Na^+$/Na
$\Delta E_0$ = 5.87 +/- 2.26, $S_0^2$ = 0.801 +/- 0.684, R-factor = 0.1113

| Bond | N | R, Å | Sigma squared $\sigma^2$, $10^{-3}$ Å$^2$ |
|---|---|---|---|
| Cu-O | 6 | 1.963 | 18.4 |
| Cu-TM | 6 | 2.890 | 8.38 |
| Cu-Na | 6 | 3.080 | 65.0 |

**b-16.** The best fit parameters for the *ex situ* Ti-NFMC **discharged to 2.0 V** vs. $Na^+$/Na
$\Delta E_0$ = 9.24 +/- 1.58, $S_0^2$ = 0.667 +/- 0.160, R-factor = 0.0184

| Bond | N | R, Å | Sigma squared $\sigma^2$, $10^{-3}$ Å$^2$ |
|---|---|---|---|
| Cu-O | 6 | 1.964 | 7.32 |
| Cu-TM | 6 | 2.956 | 6.73 |
| Cu-Na | 6 | 3.149 | 56.3 |

1 Levit, O. *et al.* Interphases Formation and Analysis at the Lithium–Aluminum–Titanium–Phosphate (LATP) and Lithium–Manganese Oxide Spinel (LMO) Interface during High-Temperature Bonding. *Energy Technology* **8**, 2000634, doi:https://doi.org/10.1002/ente.202000634 (2020).

2 Keefe, A. S., Buteau, S., Hill, I. G. & Dahn, J. R. Temperature Dependent EIS Studies Separating Charge Transfer Impedance from Contact Impedance in Lithium-Ion Symmetric Cells. *Journal of The Electrochemical Society* **166**, A3272, doi:10.1149/2.0541914jes (2019).
